# Harnessing macrophage signaling pathways and scalable engineering for next-generation immunotherapies

**DOI:** 10.1038/s41392-026-02907-x

**Published:** 2026-08-18

**Authors:** Daniela Paasch, Kathrin Haake, Chloe Kan, Cristiana Ulpiano, Eirini Nikolouli, Mi-Sun Jang, Mark-Christian Klassen, Michael Epstein, Ryohichi Sugimura, Nico Lachmann

**Affiliations:** 1 Department of Pediatric Pneumology, Allergology and Neonatology, Hannover Medical School, Hannover, Germany; 2 Evotec International GmbH, Göttingen, Germany; 3 The University of Hong Kong, Hong Kong, China; 4 InnoHK Centre for Translational Stem Cell Biology, Hong Kong, China; 5 Evotec (UK) Ltd., Abingdon, UK; 6 Biomedical Research in Endstage and Obstructive Lung Disease Hannover (BREATH), German Center for Lung Research (DZL), Hannover Medical School, Hannover, Germany; 7 Cluster of Excellence RESIST (EXC 2155), Hannover Medical School, Hannover, Germany; 8 Fraunhofer Institute for Toxicology and Experimental Medicine ITEM, Hannover, Germany

**Keywords:** Cancer therapy, Immunotherapy, Haematopoietic stem cells, Innate immune cells, Translational research

## Abstract

Macrophages have emerged as promising candidates for cell-based immunotherapies due to their intrinsic plasticity, tissue-infiltrating capabilities, and central roles in orchestrating immune responses. Their phenotypic and functional diversity within tissues and the tumor microenvironment has driven interest in harnessing these cells for therapeutic purposes. Integrating recent knowledge in signaling cascades balancing the activity of macrophages in combination with genetic engineering have enabled the development of macrophages with improved functions, including the introduction of synthetic receptors such as chimeric antigen receptors (CARs). These superior macrophages orchestrate innate immune activity with antigen-specific targeting, offering distinct advantages over conventional CAR-T and CAR-NK cell therapies, especially in solid malignancies. Emerging preclinical and early clinical data support the feasibility, safety, and therapeutic potential of macrophage-based strategies. However, successful clinical translation requires overcoming key challenges in standardization, scalable manufacturing and regulatory compliance of cell products. This review integrates current knowledge in the diversity of macrophage signaling which feeds into engineering techniques, therapeutic applications, and manufacturing innovations. Leveraging concepts of macrophage tissue plasticity highlights the potential of macrophages as next-generation cell therapeutics with broad potential in oncology and beyond, bridging fundamental immunology with translational medicine. Continued interdisciplinary research will accelerate clinical adoption and expand indications across diseases.

## Introduction

Macrophages **(**MΦs) belong to the group of white blood cells as an essential cellular component of the innate immunity which are distributed across the majority of tissues in the body.^1^ They play a crucial role as first line host defense against numerous pathogens such as bacteria, fungi and viruses, primarily via the process of phagocytosis, the engulfment and degradation of pathogens, debris and apoptotic cells. In addition to their role in tissue clearance and homeostasis, MΦs actively contribute to immune surveillance by expressing pattern recognition receptors (PRRs), enabling the detection of pathogen-associated molecular patterns (PAMPs) and damage-associated molecular patterns (DAMPs) during microbial invasion or tissue damage.^2^ This sensing capability initiates inflammatory signaling cascades and the secretion of a diverse repertoire of cytokines and chemokines that coordinate respective immune responses and recruit additional effector cells. Beyond their immunological functions, MΦs support tissue repair and wound healing via mechanisms including extracellular matrix (ECM) remodeling and the induction of angiogenesis. Taken together, these diverse functions highlight the central role of MΦs not only in host defense but also in maintaining tissue integrity under physiological and pathological conditions.

Over the last thirty years, MΦs have shifted from being viewed merely as phagocytes to being recognised as highly plastic immune cells central to tissue homeostasis, repair, and immune response orchestration. Classical MΦs are highly heterogeneous, originating from bone marrow-derived circulating blood monocytes that infiltrate tissues post-natally, mainly in response to injuries or inflammatory signals. Alternatively, tissue resident macrophages (TRMs) originate from embryonic progenitors such as the Erythroid-Myeloid-Progenitor (EMPs), derived from the yolk sac (YS) or the fetal liver that further colonize developing tissues and give rise to long-living TRMs, able to maintain their cell pool independent of infiltrating monocytes.^3^ TRMs represent specialised, sessile populations that reside in virtually all organs and acquire unique transcriptional and functional identities shaped by their microenvironment.^4^ This dual origin of MΦs adds an additional layer of complexity to their functional diversity and has important implications for both physiological processes and therapeutic interventions.

Examples of TRMs include microglia, alveolar macrophages (AMs), Langerhans cells, osteoclasts, and Kupffer cells. TRMs are shaped by the local tissue environmental with tissue-specific functions, resulting in specialized phenotypes such as alveolar MΦs in the lungs with the ability to maintain lung homeostasis, which include clearance of inhaled substances, materials, particles and pathogens as well as degradation of lung surfactant.^5^ Among others, subsets of TRMs also include microglia in the brain, contributing to brain development and neuroprotection; and Kupffer cells in the liver, crucial for liver homeostasis and regeneration. These examples illustrate how local environmental cues such as cytokines, metabolic factors, and stromal interactions imprint MΦ identity and function, enabling these cells to adapt to the specific needs of each tissue. At the same time, this context-dependent specialization contributes to their involvement in a wide range of diseases, including cancer, fibrosis, and chronic inflammatory disorders, where MΦs can either promote resolution or exacerbate pathology.

To this end, MΦ function is increasingly understood as the result of interconnected signaling, metabolic, transcriptional, and environmental programs that can be systematically decoded and therapeutically manipulated. In this context, recent advances in multi-omics, metabolic pathway analysis, single-cell technologies, and artificial intelligence-informed modelling have begun to provide a more integrated view of how MΦs adapt across tissues and disease states. These approaches enable the identification of regulatory nodes that control inflammatory activation, antimicrobial activity, tissue repair, fibrosis, or immune suppression, and thereby inform strategies to reprogram MΦs with greater precision. Consequently, MΦ-directed therapies are no longer limited to broadly modulating inflammation, but increasingly aim to engineer defined cellular functions for specific pathological environments. This includes ex vivo manipulation, genetic and metabolic engineering, in situ reprogramming, and scalable manufacturing approaches that together support the therapeutic use of MΦs across cancer, infection, fibrosis, cardiovascular, neurological, and autoimmune diseases.

As a consequence, recent knowledge into the unique function of these cells in health and disease have informed the design of next-generation MΦ-based therapies further introduced in the review below.

In particular, advances in cellular engineering, immunotherapy, and scalable manufacturing have intensified interest in harnessing MΦs as therapeutic agents, including ex vivo–modified MΦs, genetically engineered MΦs, and chimeric antigen receptor MΦs (CAR-Ms). Despite encouraging preclinical and early clinical findings, successful translation remains challenged by the intrinsic plasticity of MΦs, their context-dependent behavior within complex tissue environments, and the need for robust, standardized production platforms. This review therefore integrates current insights into MΦ development, signaling, metabolism, disease-associated plasticity, therapeutic engineering, and manufacturing strategies. By linking fundamental MΦ biology with emerging translational concepts, we provide a framework to understand how MΦ heterogeneity and environmental cues shape therapeutic outcomes and how these principles can be exploited for next-generation MΦ-based therapies.

## Insights into macrophage ontogeny to inform human macrophage generation

Historically, van Furth and colleagues proposed that tissue MΦs were short-lived and continuously replenished by bone-marrow-derived haematopoietic stem and progenitor cells (HSPCs).^6,7^ This view was challenged by early observations of MΦs arising prior to definitive haematopoiesis.^8,9^ A series of fate-mapping studies later established that many TRMs, including microglia, Langerhans cells, and AMs, originate during embryogenesis and persist also by self-renewal independently of adult HSCs.^10–17^ Much of our understanding stems from murine in vivo lineage-tracing studies. Early dye-labelling studies were replaced by sophisticated inducible Cre systems in genetically modified mice. Key fate-mapping models include Runx1^CreERT^^17^ and Csf1r^MeriCreMer^,^11^ which revealed YS haematopoiesis; as well as Cxcr4^CreERT^,^18^ which contributed to the understanding of MΦ migration; and Cx3cr1^Cre^/ Cx3cr1^CreERTss^, which helped trace TRM precursors across tissues. These approaches were crucial for distinguishing MΦs of distinct developmental origins. Mouse models remain indispensable due to ethical restrictions on human embryonic research and the accessibility of genetic tracing tools. However, technological advances now increasingly allow insights into human embryonic haematopoiesis. Of note, insights into the developmental trajectories and signaling cascades of MΦ development are crucial, as these insights inform efforts in the proper manufacturing of these cells for therapeutic purposes.

### Murine embryonic haematopoiesis

Embryonic haematopoiesis remains complex due to rapid morphological changes and dynamic tissue interactions. Current consensus divides embryonic haematopoiesis into three temporally overlapping waves^19^: primitive, erythro-myeloid progenitor (EMP) or transient-definitive wave and the definitive HSC wave.

**Fig. 1:**
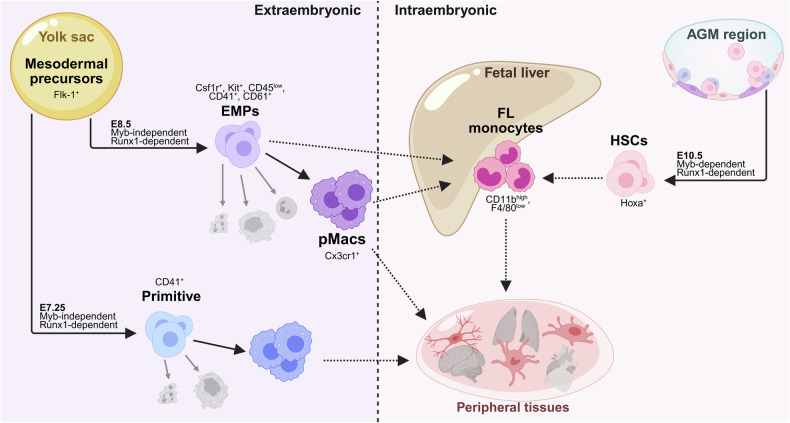
Ontogeny of macrophage and monocyte lineages during murine embryogenesis. Schematic representation of the murine extraembryonic and intraembryonic hematopoietic waves giving rise to embryonic MΦ populations. Yolk sac–derived progenitors generate primitive MΦ and erythro-myeloid progenitors that contribute to fetal liver monocytes and tissue-resident MΦs, while definitive hematopoietic stem cells emerge from the AGM region and expand in the fetal liver before seeding peripheral tissues

The mouse YS forms around E6.5^20^ and is the first site of blood formation (Fig. 1). Mesodermal precursors exposed to BMP4 and WNT signalling generate haemangioblastic cells capable of both endothelial and haematopoietic differentiation.^21,22^ These clusters are marked by Flk-1/KDR expression^23,24^ and were termed blood islands.^25–27^

The primitive wave arises around E7.25.^28–31^ It generates primitive erythrocytes, megakaryocytes,^29,32,33^ and early MΦ-like cells; although direct in vivo MΦ potential in mice is limited, in vitro and zebrafish studies support the presence of primitive MΦs.^19,34^ This is further corroborated by early microglia presence at E8.^10,35^ Primitive erythrocytes are large, nucleated, express embryonic haemoglobins,^30,36^ and arise independently of Runx1.^37,38^ Between E8.5 and E10.5, EMPs emerge through Runx1-dependent endothelial-to-haematopoietic transition (EHT).^39,40^ They are more multipotent than primitive progenitors and generate erythrocytes, platelets, MΦs, granulocytes, and mast cells.^19,29,41^ EMPs (Csf1r^+^Kit^+^CD45^low^) give rise to Cx3cr1^+^ pre-macrophages (pMacs)^15^ and after the onset of circulation at ~E9.0, they seed the fetal liver where they expand and differentiate.^8,35^ YS-derived haematopoiesis is Myb-independent, as shown in Myb^−/−^ embryos that still generate YS-derived MΦs.^11,42^ Early markers for this process include CD41 (Itga2b) and CD61 (Itgb3).^43–45^

The third wave originates intra-embryonically in the aorta-gonad-mesonephros (AGM). Hemogenic endothelial cells in the dorsal aorta begin producing HSCs around E10.5.^41,46^ Through EHT, endothelial cells generate intra-aortic haematopoietic clusters.^47^ These HSCs later migrate to the fetal liver to proliferate and mature. Unlike YS-derived progenitors, AGM-derived HSCs have long-term multilineage capacity^14,42^ and depend on Myb.^48,49^ The expression of HOXA genes distinguishes AGM-derived progenitors from other more primitive cells.^50,51^ Interestingly, YS-derived MΦs aid HSCs emerge in the AGM by providing pro-inflammatory cues; disrupting their migration (e.g. via Cx3cr1-Cx3cl1 axis) reduces HSC output.^52^ After expansion in the fetal liver, HSCs colonise the bone marrow, which becomes the lifelong site of haematopoiesis. Here, stromal cells and extracellular matrix components maintain HSC quiescence, self-renewal, and differentiation.^53,54^ Classically, HSCs differentiate through a hierarchical model. However, scRNA-seq studies have replaced this view with a more continuous, non-linear model, highlighting heterogeneity in early progenitors and biased lineage priming.^55^ Monocytes derived in adulthood circulate and differentiate into MΦs upon entering tissues. These monocyte-derived macrophages (MDMs) contribute to inflammation, repair, and, under certain circumstances, replenish TRMs.

### Human embryonic macrophage development

While mouse models have provided most knowledge of MΦ ontogeny, translating findings to humans has been limited by restricted access to embryonic tissue. Historically, human studies relied on small numbers of surgically obtained embryos, yet, they established broad parallels between mouse and human developmental waves. Carnegie staging (CS) provides morphological criteria to align human and mouse development and can be broadly mapped onto post-fertilization days.^56^ The human YS forms at CS4 and is replaced by the secondary YS at CS6, persisting until CS23.^57^ By day 16 ( ~ E7.5 equivalent), YS mesoderm thickening resembles early blood islands.^58^ Primitive erythroid, megakaryocytic,^59^ and later granulocyte-MΦ progenitors were reported at this stage.^60,61^ By 4.5 weeks, multipotent colony-forming unit (CFU) populations with burst-forming unit-erythroid (BFU-E), colony-forming unit-erythroid (CFU-E) and colony-forming unit–granulocyte-macrophage (CFU-GM) capacity were identified.^62^ The transition to fetal-liver haematopoiesis mirrors globin switching from embryonic to fetal haemoglobins.^36^ Haematopoietic potential in the para-aortic splanchnopleura appears around day 19,^63^ followed by definitive HSC emergence between days 27–35.^64^ Functional long-term HSCs were first observed at CS14,^65^ followed by fetal-liver colonisation after CS17. CD34^-^ myeloid progenitors appear by day 23, followed by CD34^+^ cells from day 30.^64^ Bone marrow haematopoiesis is only established by ~11 post-conception week (PCW), with postnatal marrow becoming the definitive HSC niche.^66^ Recent single-cell transcriptomics from elective terminations has transformed the field. The earliest atlas at CS7 mapped early YS, rostral, and caudal cell types and revealed endothelial, megakaryocyte-erythroid, and EMP-like progenitors,^67^ suggesting human YS haematopoiesis progresses earlier than in mice. A focused study of CD45^+^/CD235^-^ cells (CS11-CS23) revealed a primitive MΦ population in the CS11 YS that seeds early microglia.^68^ These cells expressed CDH5 and HBE1 alongside MΦ markers such as CD163 and MRC1. A distinct YS-derived myeloid-biased progenitor (CS11-CS13), transcriptionally similar to mouse EMPs, exhibited strong myeloid priming (MYB, MPO, LYZ) and differentiated through a monocyte-like intermediate. Building on these findings, multiple single-cell atlases have mapped haematopoiesis across the YS, AGM, and fetal liver,^50,67–73^ revealing the appearance of HOXA-expressing HSPCs in the fetal liver by CS17. Two transient YS-derived MΦ populations were described: a microglia-like population (P2RY12) interacting with neural crest cells, and a pro-angiogenic MΦ population expressing IL1B and VEGFA that dominated developing organs between CS11-CS23.^69^ Haematopoietic clusters in the dorsal aorta emerge at CS13 and express stemness genes (RUNX1, HLF, HOXA9),^50,68^while concomitantly displaying early lymphoid and myeloid lineage biases within HSCs (IL7R/IL2RG or MRC1/CX3CR1).^50^ A recent comprehensive YS atlas by Goh & Botting et al.,^70^ integrating multiple of these datasets,^71,74^ highlighted striking interspecies differences: human YS endoderm expresses EPO and THPO from 3 PCW - earlier than in mice, where these signals arise later in fetal liver. Additionally, YS stromal cells produced KITLG and NOTCH ligands predicted to support early HSC development, although signalling diverged from later bone-marrow-specific cues. By 6-8 PCW, YS stromal support wanes, with EPO production shifting to hepatocytes. Retinoic-acid and lipid-metabolism programmes are active earlier in the YS and diminish around 7 PCW.

### Macrophages derived from human iPSCs

The discovery of induced pluripotent stem cell (iPSC) technology by Takahashi and Yamanaka marked a turning point in regenerative medicine. Their landmark study demonstrated that the introduction of four transcription factors, Oct4, Sox2, Klf4, and Myc, could reprogramme murine fibroblasts to pluripotency.^75^ This was soon followed by the reprogramming of human somatic cells.^76^ Although subtle epigenetic differences persist, murine iPSCs and ESCs are highly similar in gene expression and developmental potential.^77,78^ iPSC technology has since enabled the controlled differentiation of numerous cell types, including cardiomyocytes,^79^ pancreatic β cells,^80,81^ dopaminergic neurons,^82^ MΦs,^83^ and erythrocytes.^84^ Improvements such as xeno-free culture, three-dimensional differentiation, and directed lineage specification have contributed to increasingly robust and standardised MΦ differentiation protocols. Although protocols differ, iPSC-derived macrophage (iMac) differentiation broadly mimics embryonic haematopoiesis. BMP4 is commonly used to induce haematopoietic mesoderm through regulation of GATA-1 and SCL^85^ and also contributes to HSC maintenance.^86^ VEGF-A activates Flk-1 on mesodermal progenitors and promotes endothelial specification.^87^ IL-3 supports EHT and early commitment via MAPK/STAT3 signalling.^88^ Small molecules modulating WNT and Activin/Nodal pathways, including CHIR99021 and Activin A, promote mesoderm formation, while the withdrawal or inhibition of these pathways supports hemogenic endothelium (HE) differentiation towards myeloid lineages.^89,90^ Additional early haematopoietic regulators, such as SCF,^91^ IL-6, FGF2, and thrombopoietin, are commonly included, whereas macrophage colony-stimulating factor (M-CSF) drives MΦ specification. iPSC technology has also enabled the generation of organoids - self-organising, three-dimensional tissues modelling organ morphology and function. Organoids resembling brain,^92^ kidney,^93^ and intestine^94^ have been used for developmental and disease modelling studies. Nevertheless, many iPSC-derived cell types retain fetal characteristics. For example, iPSC-derived hepatocytes show reduced metabolic activity and variable engraftment compared to adult hepatocytes,^95^ highlighting the challenge of achieving full maturation of the produced cell types. Recent work has focused on haematopoietic organoids (“Hemanoids”), which recapitulate YS haematopoiesis and efficiently produce MΦs.^88,89,96–98^ These organoids generate mesoderm expressing KDR,^89^ followed by endothelial populations marked by CDH5 and PECAM. A subset transitions to hemogenic endothelium expressing RUNX1, with ERG, FLI1, and MEIS1 contributing to EHT regulation.^99,100^ Several studies highlight parallels to embryonic development: MΦs arise independently of Myb, as shown in both murine YS studies^11^ and MYB-knockout iPSC organoids.^98^ Conversely, RUNX1- or SPI1-deficient iPSCs fail to generate haematopoietic cells, and erythroid cells exhibit predominantly primitive haemoglobin Hbε.^89,96^As direct study of human embryogenesis is limited, iPSC-derived systems offer an essential platform for modelling early haematopoiesis, enabling detailed investigation of YS-like MΦ development and the origins of HSC-independent immune lineages.

## The general concept of macrophage plasticity and polarization: signaling pathways driving functional diversity

While developmental origin shapes MΦ identity and tissue residency, it alone cannot explain the wide functional diversity observed across tissues and disease settings. To fulfil this diverse function, MΦs display a profound tissue plasticity which is mediated by their ability to adopt towards a wide range of functionally-distinct phenotypes in response to microenvironmental signals. This concept is traditionally introduced by the simplified M1/M2 paradigm: Classically-activated (often referred as M1) MΦs arise in response to pro-inflammatory signals like interferon-gamma (IFN-γ) and microbial components such as lipopolysaccharide (LPS) acting through Toll-like receptor 4 (TLR4). These stimuli initiate signaling through MyD88-dependent pathways, leading to activation of NF-κB, MAPKs (p38, JNK, ERK) and STAT1. Pro-inflammatory MΦs are further characterized by a distinct metabolic profile, primarily utilizing the glycolytic pathway, resulting in a rapid increase in glucose uptake and glycolytic flux, facilitating accelerated ATP generation and providing essential intermediates for biosynthetic processes.^101^ This metabolic shift is closely linked to the expression of pro-inflammatory genes such as IL-1β, TNF-α, iNOS, CXCL10, IL-6, and IL-12, promoting antimicrobial activity and T helper 1 (Th1) immune responses. Additionally, these cells are characterized by an active pentose phosphate pathway (PPP), a restructured tricarboxylic acid (TCA) cycle, fatty acid synthesis, nitric oxide production, and pronounced redox activity.^102^ In contrast, alternatively-activated (often referred as M2) MΦs are induced by anti-inflammatory signals like interleukin-4 (IL-4) and interleukin-13 (IL-13), signal through the IL-4 receptor alpha chain activating STAT6 that further drives the transcription of genes involved in tissue repair, matrix remodeling and immunosuppression including Arg1, Mrc1 (CD206) and Retn1a. Anti-inflammatory (M2) MΦs further rely on oxidative phosphorylation (OXPHOS), characterized by elevated mitochondrial respiration and efficient ATP synthesis, alongside enhanced fatty acid oxidation and arginase-dependent arginine metabolism. This metabolic program supports IL-10–driven signaling through STAT3, together with TGF-β/SMAD pathways, thereby reinforcing anti-inflammatory functions, promoting wound healing, and maintaining tissue homeostasis.^103^ However, accumulating transcriptomic and single cell data have revealed that MΦs in vivo rarely conform to these two stages of activation.^104,105^ Instead, they exhibit a continuum or mosaic of activation states that integrate inflammatory, metabolic, and stromal cues from their microenvironment. For instance, tumor-associated macrophages (TAMs) frequently co-express canonical pro- (M1) and anti-inflammatory (M2) markers, reflecting dynamic reprogramming in response to fluctuating cytokine gradients, hypoxia, and metabolic stress. Similarly, in chronic inflammatory diseases, MΦ phenotypes can transition reversibly between pro- and anti-inflammatory states depending on disease stage and tissue context. Recent integrative analyses propose multi-dimensional classification frameworks based on transcriptional, metabolic, and functional profiling, offering a more accurate representation of MΦ heterogeneity in health and disease.^103^ Consequently, while the M1/M2 paradigm provides a useful conceptual framework, MΦs in tissues exist along a spectrum of intermediate and context-dependent activation states. This plasticity enables adaptive immune responses but can also contribute to disease progression. A detailed understanding of the molecular drivers and signaling pathways underlying MΦ heterogeneity is therefore essential for the rational design of targeted immunotherapies across diverse pathological conditions.

## Macrophage plasticity in disease: context-dependent roles and therapeutic opportunities

The context-dependent nature of MΦs activation becomes particularly evident in disease, where local tissue microenvironments and disease-specific cues drive MΦ functions towards either exerting beneficial roles in host defense, tissue repair, and resolution of inflammation or promoting pathology by sustaining chronic inflammation, fibrosis, or immune suppression. Consequently, disease-associated MΦ states have emerged as both critical determinants of disease progression and attractive targets for therapeutic intervention for numerous diseases, including cardiovascular, neurodegenerative, infectious, and autoimmune disease, as well as cancer. Understanding how these functional states arise and operate across pathological settings is essential for determining when MΦs should be targeted or reprogrammed in vivo, or alternatively manipulated ex vivo and administered as cell therapies. In this section, selected disease contexts are discussed to illustrate how MΦ functional plasticity shapes disease pathophysiology and how it can be therapeutically exploited through MΦ-directed interventions.

### Macrophage-based therapies for cardiovascular repair

The importance of MΦ plasticity is particularly evident in cardiovascular disease (CVD), where MΦs orchestrate both tissue injury and repair in response to acute and chronic inflammatory cues. In CVD, MΦs are rapidly recruited to sites of tissue injury and exert pleiotropic functions, including inflammatory signalling, debris clearance, interaction with stromal and vascular cells, and regulation of extracellular matrix (ECM) remodelling and angiogenesis. Depending on disease context and stage, these activities can either support tissue repair or drive pathological inflammation and fibrosis.^106^ Myocardial infarction (MI) exemplifies an acute setting in which precise temporal control of MΦ phenotype is critical for effective tissue repair. Accordingly, cytokine-based strategies have been developed to promote reparative MΦ functions following MI.^107,108^ Jung et al. demonstrated that timed IL-10 infusions inhibit post-MI inflammation and reduce collagen deposition by promoting anti-inflammatory MΦ polarization.^108^ Similarly, intramyocardial delivery of IL-10 combined with NF-κB inhibitor (SN50) loaded in a supramolecular hydrogel showed improved cardioprotective effect in a rat model of MI, compared to intravenous administration. A related approach involved the on-demand delivery of IL-4-pDNA nanocarriers at the infarct region in vivo, using a matrix-metalloproteinase (MMP)-responsive hydrogel system, which promoted anti-inflammatory polarization, reducing cardiac inflammation and myocardial fibrosis while enhancing angiogenesis.^107^ In addition, pharmacological modulation of MΦ polarization has also shown therapeutic potential, as Dapagliflozin and Pioglitazone promote anti-inflammatory polarization, attenuate post-MI inflammation, and alleviate myocardial fibrosis.^109,110^ Beyond in vivo modulation, in vitro reprogramming strategies allow greater control over MΦ phenotype before transplantation, minimize off-target effects, and potentiate the MΦ reparative action post-MI. In one study, bone marrow mononuclear cells (BM-MNCs) conditioned with M-CSF and IL-4 were transplanted in mice following MI, improving microvascular formation, reducing cardiomyocyte hypertrophy, and attenuating fibrosis after MI, while showing enhanced survival and stimulating resident MΦs toward an anti-inflammatory phenotype.^111^ Similarly, transplantation of hypoxia-primed MΦs improved cardiac function and ventricular structure, reduced scar formation and fibrotic remodelling, and enhanced angiogenesis and cardiomyocyte proliferation.^112^ Recent reports have identified genetically engineered MΦs as promising regenerative agents after MI.^113^ For example, Tan et al., engineered murine bone marrow-derived MΦs (BMDMs) to overexpress C-C chemokine receptor type 2 (CCR2) and MerTK, a significant contributor to cardiac repair after MI, and chemically decorated with a CD47 signal antagonist. Following intravenous injection in a mouse myocardial ischemia–reperfusion (MI/R) model, these MΦs selectively migrated to the injured myocardium, restored efferocytosis, reduced inflammation, and preserved cardiac function.^113^ Moreover, chimeric antigen receptor (CAR) technology, which is discussed in detail later in this review, has also been applied to enhance MΦ therapeutic functions. In the context of CVD, fibroblast activation protein (FAP)-targeting CAR macrophages (CAR-Ms) generated ex vivo or in vivo have been shown to selectively phagocytose FAP-positive activated fibroblasts, resulting in reduced cardiac fibrosis and improved cardiac function in MI/R injury models.^114,115^

While post-MI injury represents an acute setting in which temporal control of MΦ phenotype is critical, MΦ-driven mechanisms also underlie chronic cardiovascular pathologies such as atherosclerosis (AS). Upon endothelial dysfunction, MΦs infiltrate the subendothelial layer, take up modified low-density lipoprotein (LDL), and form foam cells, driving plaque progression through sustained inflammation, ECM remodelling, and necrosis.^116^

Therapeutic strategies have therefore focused on limiting inflammatory monocyte-derived MΦ recruitment,^117^ including CCL2 inhibition through adenoviral delivery of an N-terminal deletion mutant of the CCL2 gene, and CCR2 antagonism using a small molecule termed 15a, with both methods reducing plaque MΦ content and improving lesion stability in murine models.^118,119^

Beyond limiting MΦ recruitment, targeting MΦ lipid handling has been explored to prevent foam cell formation and early plaque growth. For instance, Mäkinen et al. have demonstrated that short hairpin RNAs (shRNAs)-mediated silencing of MΦ scavenger receptor A, important for the uptake of modified lipoproteins, significantly reduced foam cell formation in vitro and led to a significant decrease of the atherosclerotic lesion area in vivo.^120^ Additional approaches include miRNA-33 inhibition to enhance cholesterol efflux and limit lipid accumulation,^121^ cytokine modulation such as IL-19 to suppresses MΦ inflammation and maintain cholesterol homeostasis in atherosclerotic lesions.^122^ In addition, targeting defective efferocytosis, Yao and colleagues developed biomimetic “zombie MΦs” (termed ZARMs) that deliver anti-inflammatory cargo to atherosclerotic plaques. ZARMs are generated by cryo-shock-based encapsulation of reactive oxygen species (ROS)-responsive nanoparticles loaded with atorvastatin into living MΦs. Following intravenous administration in atherosclerotic mice, ZARMs preferentially accumulated in ROS-enriched plaques, reduced CD47 expression on foam cells, enhanced efferocytosis, and attenuated plaque progression.^123^

### Macrophage-targeted therapies in neurodegeneration

Beyond peripheral tissues, MΦ plasticity critically influences disease progression in the central nervous system (CNS), where resident microglia and infiltrating monocyte-derived MΦs regulate inflammatory and reparative responses in a highly compartmentalized microenvironment. Chronic neuroinflammation, neuronal loss, and disrupted tissue homeostasis define neurodegenerative diseases, with myeloid cells playing central roles.^124^

In Alzheimer’s disease (AD), characterized by amyloid-β (Aβ) deposition, synaptic dysfunction, and cognitive decline, microglia/MΦs contribute to both pathology and repair, making them attractive therapeutic targets. Strategies to enhance MΦ-mediated Aβ clearance include inhibition of Smad3 signaling using SIS3, which boosted peripheral MΦ-driven Aβ clearance in AD mouse models, reducing amyloid deposition, neuroinflammation, and cognitive deficits without directly activating brain microglia.^125^ Complementary approaches, such as activation of G protein-coupled receptor GPR34 to increase microglial uptake of fibrillar Aβ and soluble colony-stimulating factor-1 receptor (CSF1R)-based strategies to promote microglial recruitment and clustering around plaques, also reduced amyloid burden and neuritic dystrophy in AD models.^126,127^ In parallel, engineered MΦ-based therapies are emerging as promising approaches for AD treatment. Beyond amyloid clearance, modulation of chronic microglia-driven neuroinflammation has been widely explored. Inhibition of the NLRP3 inflammasome using small-molecule inhibitors such as MCC950 or JC-124 reduced microglial activation, pro-inflammatory cytokine release, and synaptic loss, while improving cognitive outcomes in AD mouse models.^128,129^

In contrast to AD, Parkinson’s disease (PD) is characterized by intracellular protein aggregation, selective neuronal vulnerability, and chronic neuroinflammation, with microglia/MΦs influencing inflammation, protein clearance, and neuronal survival. PD involves progressive dopaminergic neurons (DA) loss in the substantia nigra (SN), misfolded α-synuclein (α-syn) accumulation, and sustained microglial activation.^124^ Therapeutic strategies therefore focus on limiting detrimental neuroinflammation. Agents such as melatonin promote an anti-inflammatory, M2-like microglial phenotype and protect dopaminergic neurons, improving motor symptoms in PD models.^130^ Similarly, NADPH oxidase inhibitors and NF-κB pathway inhibitors (e.g., hypoestoxide, lenalidomide) reduce pro-inflammatory microglial activation, protect dopaminergic neurons, and improve motor deficits in PD models.^131–133^

Beyond pharmacological approaches, MΦ reprogramming and cell-based therapies have shown promising results in PD. Insulin-like growth factor 2 (IGF2)-reprogrammed murine MΦS prevented α-syn–induced pro-inflammatory polarization and reduced α-syn accumulation and microglial activation in the substantia nigra.^134^ Similarly, HSPC-derived MΦs/microglia engineered to express glial cell line-derived neurotrophic factor (GDNF) selectively accumulated in degenerating nigrostriatal regions, providing sustained neurotrophic support, reducing dopaminergic neuron loss, and improving motor function without major adverse effects.^135^ Transplantation of HSPCs engineered with codon-optimized human beta-glucocerebrosidase (GBA) also ameliorated GBA deficiency–associated PD in mouse models.^136^

### Macrophage-directed therapies in autoimmune disease

In autoimmune diseases, dysregulated macrophages (MΦs) drive inflammation, tissue damage, and chronic disease, but their plasticity also allows them to promote resolution and repair. Therapeutic strategies targeting MΦ recruitment, activation, or function are therefore promising for controlling disease progression.^137^

In rheumatoid arthritis (RA), synovial MΦs are major drivers of chronic inflammation, joint destruction, and systemic disease manifestations. Pro-inflammatory MΦ populations sustain synovitis through persistent cytokine production, matrix degradation, and promotion of osteoclastogenesis, whereas anti-inflammatory MΦ states are associated with disease resolution and tissue repair.^137^ Therapeutic strategies in RA focus on reprogramming synovial MΦs towards anti-inflammatory states. In a collagen-induced arthritis (CIA) mouse model, anti-inflammatory MΦ-derived extracellular vesicles reprogrammed MΦs in vivo, reducing joint swelling, synovial inflammation, and cartilage and bone damage without systemic toxicity.^138^ A complementary nanotherapeutic approach employed mannose-functionalized polymersomes carrying hydroxychloroquine selectively targeted pro-inflammatory MΦs in a zymosan-induced RA model, promoting anti-inflammatory polarization, lowering pro-inflammatory cytokines, and improving joint pathology.^139^ Similarly, ROS/pH dual-responsive micelles delivering celastrol also accumulated in inflamed joints, reversed MΦ polarization towards an anti-inflammatory state, and suppressed synovial inflammation and fibroblast invasion in CIA rats.^140^ Beyond pharmacological approaches, engineered MΦ-based cell therapies offer an alternative strategy. Klimak et al. engineered BMDMs with an NF-κB–responsive IL-1 receptor antagonist (IL-1Ra) circuit, enabling inflammation-responsive cytokine delivery. After intraperitoneal administration in RA mice, these MΦs homed to inflamed joints, demonstrated controlled IL-1Ra production, suppressing synovial inflammation and reduced disease severity.^141^ Similarly, blocking GM-CSF signaling with antibodies (e.g., mavrilimumab) decreased MΦ infiltration, cytokine production, and joint pathology by inhibiting activation signals rather than directly reprogramming MΦs preclinical RA models.^142^

Another prevalent chronic autoimmune disorder is multiple sclerosis (MS), which is a demyelinating disease of the CNS characterized by immune-mediated myelin and axon damage, neuroinflammation, and progressive neurological decline. In MS lesions, resident microglia and infiltrating MΦs drive disease progression through myelin phagocytosis, antigen presentation, and sustained inflammation, making them attractive targets for therapeutic reprogramming.^137,143^ In experimental autoimmune encephalomyelitis (EAE) models, intravascular administration of ex vivo polarized MΦs towards an anti-inflammatory phenotype via NF-κB inhibition reduced spinal inflammation, demyelination, and disease progression.^144^ Similarly, activating the purinergic P2X4 receptor with the ivermectin favoured an anti-inflammatory, pro-repair microglia/MΦ phenotype, enhanced myelin clearance, and improved motor function in EAE mice.^145^ Alternatively, inorganic-organic hybrid nanoparticles carrying the glucocorticoid betamethasone (BMP-NPs) selectively reprogrammed MΦs in vivo, inducing anti-inflammatory polarization and reducing spinal MΦ numbers.^146^ Interestingly, PEGylated liposomes targeting MΦs also reduced IL-1β–mediated neuroinflammation and EAE severity without additional drugs.^147^ Additionally, following intravenous administration, embryonic stem cell–derived microglia expressing neurotrophin-3 engrafted into CNS lesions, promoted an anti-inflammatory environment, and supported neuronal sprouting while reducing demyelination.^148^

Furthermore, extracellular vesicles derived from engineered microglia co-expressing IL-4 and lactadherin similarly reprogrammed endogenous microglia toward an anti-inflammatory phenotype and attenuated neuroinflammation in EAE models.^149^

### Macrophage-targeted therapies in liver disease and fibrosis

Chronic inflammation can drive metabolic liver diseases, particularly metabolic dysfunction–associated fatty liver disease (MAFLD) and its inflammatory form, metabolic dysfunction–associated steatohepatitis (MASH), which are characterized by hepatic lipid accumulation, hepatocellular injury, and progression to fibrosis, cirrhosis, and hepatocellular carcinoma. Liver-resident MΦs (Kupffer cells) and infiltrating MΦs are central drivers of disease progression by regulating steatosis, inflammation, and fibrogenesis.^150^ For consistency with the literature, the terms non-alcoholic fatty liver disease (NAFLD) and non-alcoholic steatohepatitis (NASH) are used when referring to studies published prior to the adoption of the MAFLD/MASH nomenclature.^151^ Targeting MΦ immunometabolism is a promising therapeutic strategy in MAFLD/MASH. For instance, celastrol ameliorates liver inflammation in NAFLD/NASH models by binding pyruvate kinase M2 (PKM2), shifting MΦ metabolism from aerobic glycolysis toward mitochondrial oxidative phosphorylation, and promoting an anti-inflammatory phenotype, improving liver pathology.^152^ Similarly, glycyrrhetinic acid suppresses MΦ glycolysis via the STAT3–HIF-1α axis, reducing pro-inflammatory MΦ infiltration and hepatocyte apoptosis, resulting in improved pathological features in NAFLD models.^153^ Other compounds, including lapachol, annexin A5, and digoxin, also suppress pro-inflammatory MΦ activation, attenuating steatosis and liver inflammation in NASH models through modulation of PKM2-related metabolic pathways.^154–156^ Beyond metabolic reprogramming, therapeutic strategies have targeted MΦ recruitment, inflammatory signaling, and pro-fibrotic activity in MAFLD/MASH. Cenicriviroc (CVC), a dual CCR2/CCR5 inhibitor, reduced pro-inflammatory MΦ recruitment and fibrosis in preclinical models of liver fibrosis and was safe in NASH patients but failed to improve fibrosis in phase II trials.^157,158^ Alternatively, selective silencing of HMGB1 in activated hepatic MΦs using mannose-modified lipid nanoparticles delivering HMGB1-specific siRNA reduced inflammation and steatosis, promoted an anti-inflammatory MΦ phenotype, and improved liver function in NASH mouse models.^159^ Fibrosis is a common pathological outcome of chronic tissue injury in metabolic and inflammatory diseases, characterized by excessive ECM deposition, tissue stiffening, and loss of organ function.^160^ In the liver, persistent inflammatory stimuli such as MAFLD/MASH promote fibrosis and cirrhosis through sustained MΦ activation and dysregulated matrix remodeling.^161^ However, MΦs also contribute to fibrosis resolution by driving ECM remodeling, making them attractive therapeutic targets. Consistent with this, MΦ-based therapies have shown efficacy in advanced liver disease.^162^ Pouyanfard et al. demonstrated that transplantation of human iMacs significantly improved liver function, reduced inflammation, and attenuated liver fibrosis in a mouse xenograft model, with anti-inflammatory-polarized MΦs showing superior effects.^163^ Similarly, Yao et al. demonstrated that IGF2-primed BMDMs reduced liver inflammation and fibrosis, improved liver function, and promoted hepatocyte regeneration in cirrhotic mice by upregulating NR4A2 and inducing an anti-inflammatory, matrix-remodeling phenotype.^164^

### Macrophage-based antifibrotic strategies in lung and kidney disease

While the liver provides a classic example of MΦ-driven fibrotic progression and therapeutic targeting, analogous MΦ-dependent mechanisms operate across multiple organs, including the lung and kidney, where fibrosis drives progressive organ dysfunction. In the lung, idiopathic pulmonary fibrosis (IPF) is a progressive interstitial disease characterized by excessive ECM accumulation, alveolar remodelling, and irreversible loss of lung function. MΦs drive fibrotic progression through profibrotic signalling, fibroblast activation, and sustained cytokine secretion that promote collagen deposition.^165^ Consequently, targeting MΦ function has shown promising antifibrotic effects in preclinical models. In bleomycin-induced pulmonary fibrosis (PF), MΦ-targeted delivery of methyl-CpG-binding protein 2 (Mecp2) siRNA via cationic liposomes reversed established fibrosis by reducing anti-inflammatory polarization and profibrotic activity.^166^ Similarly, liposomal dexamethasone decreased MΦ infiltration and collagen deposition while limiting systemic toxicity bleomycin-induced PF models.^167^ Pharmacological activation of SHP-1 likewise reduced pathogenic MΦs, suppressed profibrotic markers, and attenuated fibrosis with improved lung architecture and survival.^168^

In the same line, administration of recombinant CXCL11 in PF mouse models shifted MΦs toward a less fibrogenic pro-inflammatory-phenotype and reduced fibrosis. Notably, the secretome from ex vivo M1-polarized MΦs similarly reversed lung fibrosis in PF mice.^169^ In parallel, MΦ-based cell therapies have shown antifibrotic potential in IPF models. Liu et al. engineered murine MΦs to secrete antifibrotic mediators (IL-10, a soluble TGF-β receptor, or CD147), each of which significantly attenuated bleomycin-induced fibrosis while remodelling the fibrotic niche, with sequential combination therapy providing superior efficacy compared with individual treatments.^170^

In the kidney, renal fibrosis, defined by excessive ECM accumulation within the interstitium and glomeruli, represents the final common pathway of chronic kidney disease (CKD) and ultimately leads to progressive loss of renal function. Both experimental animal models and human studies have demonstrated that MΦs play a key role in driving kidney injury, inflammation, and fibrotic progression.^171^ In this context, therapeutic modulation of MΦ phenotypes has emerged as a promising approach to attenuate renal fibrosis. In a rat model of CKD, treatment with the sodium-glucose co-transporter 2 inhibitor empagliflozin reduced renal interstitial fibrosis and improved renal function by suppressing the polarization of MΦ towards an M2 phenotype through regulation of mTOR and mitophagy-related pathways, thereby limiting pro-fibrotic signalling and ECM accumulation.^172^ Another pharmacological strategy involves activation of the G protein-coupled estrogen receptor 1 (GPER1) to modulate MΦ activation states. In unilateral ureteral obstruction (UUO)-induced renal fibrosis models, administration of the selective GPER1 agonist G-1 decreased MΦ infiltration and phenotypic activation, resulting in reduced tubulointerstitial fibrosis, diminished ECM expression by fibroblasts, and improved histological outcomes.^173^ Beyond MΦ reprogramming, selective depletion of pro-fibrotic MΦ subsets has also demonstrated therapeutic potential. In a study by Ouyang et al., a bioactivated in vivo assembly peptide (BIVA-PK) was developed to selectively target MΦs co-expressing the profibrotic markers Fn1, Spp1, and Arg1. BIVA-PK induced disruption of MΦ membrane integrity and mitochondrial homeostasis, leading to selective elimination of M2-like MΦs. Treatment with BIVA-PK effectively reshaped the immune microenvironment and attenuated fibrosis in a mouse model of renal injury.^174^ In addition, MΦ-based gene therapy approaches have been explored in a UUO model. BMDMs genetically engineered to overexpress neutrophil gelatinase-associated lipocalin (NGAL) reduced renal inflammation, tubular epithelial injury, and interstitial fibrosis, underscoring the potential of MΦ-mediated delivery of cytoprotective factors to indirectly limit fibrotic progression.^175^

### Harnessing macrophages for antimicrobial and antiviral therapies

Infectious diseases represent another context in which MΦs occupy a central yet paradoxical role, acting both as essential antimicrobial effector cells and, in some settings, as intracellular reservoirs that enable pathogen persistence and immunopathology.^176,177^ Consequently, therapeutic strategies that enhance MΦ antimicrobial functions, redirect their inflammatory responses, or exploit them as cellular or drug delivery vehicles have emerged as promising approaches for a broad range of bacterial and viral infections. For instance, in tuberculosis (TB), MΦs are both the primary host cells for Mycobacterium tuberculosis (Mtb) and central effectors of host immunity. Following Mtb uptake, the antimicrobial action of MΦs is dampened, allowing survival and replication of the pathogen within them.^176^ Accordingly, enhancing MΦ antimicrobial pathways can improve TB outcomes. In Bekale et al., immunomodulatory Curdlan poly(lactic-co-glycolic acid) nanoparticles treatment induces autophagy in Mtb-infected MΦs, modulates the secretion of immune cell recruitment cytokines and chemokines and reduces bacterial burden in the lungs of infected mice.^178^ In parallel, liposomal encapsulation of the protease inhibitor saquinavir significantly increased uptake by Mtb-infected human monocyte-derived MΦs and enhanced intracellular killing of both drug-susceptible and multidrug-resistant Mtb strains by overcoming pathogen-induced cathepsin proteolytic activity suppression.^179^ Overall, these studies highlight how MΦ-targeted modulation can restore antimicrobial functions in TB, a principle that has increasingly been extended to other intracellular bacterial infections. Additionally, MΦ-targeted delivery strategies have also been explored for the treatment of intracellular bacterial infections. In one study, cyclic arginine-glycine-aspartic acid (cRGD)-modified liposomal vancomycin was developed to enhance MΦ targeting and uptake via integrin-mediated phagocytosis. This approach significantly increased intracellular antibiotic accumulation, promoted rapid phagocytosis by MΦs, and improved clearance of intracellular bacteria both in vitro and in murine Methicillin-resistant Staphylococcus aureus (MRSA) infection models.^180^ Alternatively, MΦ-based strategies have also been explored for bacterial infections immunotherapies. For instance, mass production of human iPSC-derived MΦs in bioreactors was shown to rescue mice from Pseudomonas aeruginosa-mediated acute lower respiratory tract infections following intrapulmonary transplantation, reducing bacterial load and improving survival.^83^ Notably, in a recent study, human iPSC-derived MΦs exhibited enhanced innate immune responses compared with human monocyte-derived MΦs in Mtb infection models.^181^ Moreover, CAR engineering has emerged as a strategy to redirect MΦ phagocytic and bactericidal functions. Recent studies have demonstrated that nanoparticle-based delivery platforms can be used to engineer CAR-expressing MΦs in situ, while concomitantly silencing caspase-11 (CASP11) to limit inflammatory cell death and preserve MΦ effector function. This combined approach successfully enhanced Staphylococcus aureus clearance in both an MRSA-infected orthopedic implant model and a systemic MRSA sepsis mouse model.^182,183^

In addition to bacterial pathogens, MΦs play critical roles in viral infections, where they can act as antiviral effector cells, viral reservoirs, or drivers of immunopathology, depending on the pathogen and inflammatory context.^177^ In preclinical models of viral pneumonia, antibody-conjugated lipid nanoparticles targeting lung MΦs have been used to deliver siRNA against pro-inflammatory TGF-β-activated kinase 1 (TAK1), resulting in diminished pro-inflammatory cytokines TNF-α, IL-1β and IL-6 secretion, and marked amelioration of lung injury in influenza virus-infected mice.^184^ In the context of severe coronavirus infection, biomimetic MΦ membrane-coated nanocarriers loaded with the antiviral drug lopinavir were shown to neutralise key pro-inflammatory cytokines, suppress MΦ and neutrophil activation, and significantly improve survival outcomes in a mouse model of coronavirus infection.^185^ Alternatively, MΦ-based virus clearance was investigated by Fu and colleagues in which CAR engineering was used to reprogram MΦ phagocytic activity against SARS-CoV-2.^186^ The engineered human MΦs demonstrated antigen-specific clearance of SARS-CoV-2 virions in vitro without the secretion of pro-inflammatory cytokines. In chronic viral infections such as human immunodeficiency virus (HIV), MΦs contribute to viral persistence despite antiretroviral therapy, and MΦ-targeted drug delivery has been investigated as a therapeutic avenue. In Wu et al., lipid particles decorated with folate to target activated MΦs efficiently delivered the reverse-transcriptase inhibitor rilpivirine into HIV-infected MΦs. Similarly, gold nanoparticle-based MΦ-targeted carriers have been used to deliver the antiretroviral stavudine to primary human MΦs, enhancing intracellular drug uptake and potentiating antiviral activity while also promoting MΦ antiviral activation profiles.^187^ Additionally, MΦs have been exploited as cell delivery systems of antiviral agents. An early study demonstrated that murine BMDMs loaded ex vivo with nanoformulated indinavir systemically administered in a murine model of HIV-1 encephalitis (HIVE) readily migrated to areas of HIVE in the brain, achieving sustained antiretroviral release, high local drug levels, and reduced viral replication in HIVE tissues.^188^

## Harnessing metabolic regulation of macrophage function and polarization in disease

Metabolism serves not only as the energy source for cells but also functions as a regulatory system that influences cell behaviour. Many metabolic intermediates act as signalling molecules, directly affecting gene expression, epigenetics, and ultimately determining cell fate. In MΦs especially, metabolism actively shapes cell polarization and greatly contributes to their adaptability, enabling them to respond to varying environmental conditions and immune challenges. Since metabolic pathways serve as regulatory mechanisms influencing MΦ phenotypes and immune responses, they have been targeted in various experimental models to develop innovative therapeutic strategies, including infectious disease management, intervention in chronic inflammation, and approaches for cancer treatment.

### Metabolic reprogramming in macrophages in the frame of infectious diseases

MΦs are critical components of the innate immune system, playing a central role in combating infections. In recent decades, increasing attention has been given to the metabolic reprogramming of MΦs within the context of infectious diseases. Numerous viral and bacterial infections have demonstrated that severe disease progression often correlates with dysregulated cytokine production, commonly referred to as a cytokine storm. It has been suggested that targeted metabolic interventions may help modulate excessive immune responses and mitigate the detrimental consequences of cytokine storms. Agents such as dimethyl fumarate (DMF), metformin, methotrexate, rapamycin, and dexamethasone are among the drugs currently employed in clinical settings to influence metabolic pathways and prevent cytokine storms. For example, dexamethasone primarily acts on MΦs by boosting OXPHOS and inhibiting the production of inflammatory factors like CCL2 and TNF. Additionally, both in vitro experiments and in vivo mouse models have demonstrated that dexamethasone enhances the ability of MΦs to kill and engulf bacteria.^189,190^ Recently, dexamethasone has also been used to treat severe cases of COVID-19, where it increased survival rates by 20–30%.^191^ In a similar manner, the itaconate derivative 4-OI activates the anti-inflammatory transcription factor Nrf2, leading to reduced cytokine production and modulation of type I interferon responses, which fosters an anti-inflammatory state during acute infections.^192^ Recent research has advanced our understanding of metabolic interventions in MΦs infected with Mtb. In a proof-of-concept study, Yadav et al. demonstrated that treatment with meclizine facilitated metabolic reprogramming by shifting cellular metabolism from OXPHOS to glycolysis. This shift led to elevated ROS levels and decreased Mtb drug tolerance.^193^ Another study explored the role of fatty acid metabolism in limiting Mtb replication. By employing CRISPR-Cas9 gene knockouts in infected MΦs, researchers identified several fatty acid metabolism-associated genes—including SLC27A1, PLIN2, and CPT2—whose deletion resulted in significant restriction of intracellular Mtb growth.^194^ Additionally, exogenous inhibitors targeting fatty acid transport and β-oxidation, such as the FATP1 inhibitor trimetazidine, were found to further reduce intracellular Mtb proliferation when applied to infected MΦs.^194^

### Metabolic modulation of macrophages in the context of pro-inflammatory disease management

Pro-inflammatory diseases constitute a considerable portion of global morbidity, with their incidence continuing to rise internationally. Current therapeutic strategies are often inadequate, underscoring the potential of modulating cellular metabolic pathways as a novel approach to treating pro-inflammatory disorders. MΦs are now recognized as key drivers of pathology in a wide range of pro-inflammatory conditions and as attractive targets for therapeutic intervention. Of particular note, elevated expression of the glycolytic regulator 6-phosphofructo-2-kinase / fructose-2,6-bisphosphatase 3 (PFKFB3) has been documented in patients with RA. Experimental genetic knockdown of PFKFB3 in murine MΦs resulted in reduced pro-inflammatory polarization and diminished secretion of key cytokines, including IL-1β, IL-6, and TNFα. Furthermore, in a murine model of RA, partial knockdown of PFKFB3 resulted in an improved disease phenotype, suggesting that inhibition of glycolysis may represent a viable therapeutic strategy.^195^ A notable example of a drug capable of modulating MΦ metabolism and already approved for the treatment of multiple sclerosis and psoriasis is the fumaric acid ester dimethyl fumarate (DMF).^196^ Upon administration, DMF is rapidly metabolized to monomethyl fumarate and demonstrates anti-inflammatory effects. Mechanistically, DMF activates the NRF2 antioxidant pathway via electrophilic modification of KEAP1 and directly inhibits metabolic enzymes such as succinate dehydrogenase and glycolytic regulators. These actions result in decreased succinate-driven HIF-1α signalling, reduced inflammasome activation, and diminished production of pro-inflammatory cytokines.^197^ Additionally, DMF has been investigated in experimental mouse models for the treatment of autoimmune encephalomyelitis^198^ and Duchenne muscular dystrophy.^199^ The clinical efficacy of DMF demonstrates that targeting immune cell metabolism is a viable strategy for managing chronic inflammatory diseases and has contributed to the recognition of metabolic reprogramming of MΦs as a potential approach for developing advanced anti-inflammatory therapies.

### Metabolic modulation of macrophages as a strategy for cancer therapy

Especially in solid tumours, metabolic reprogramming of TAMs offers a potential strategy to alter the tumour microenvironment toward a more pro-inflammatory and anti-cancer state, thereby enhancing the efficacy of current therapeutic approaches. One approach was shown by Freemerman et al., demonstrating stable overexpression of the glucose transporter 1 (GLUT1) in RAW264.7 MΦs, leading to increased glucose uptake and metabolism, elevated levels of pentose phosphate pathway intermediates, and reduced cellular oxygen consumption rates. This genetically induced increase in glucose utilization prompted a reactive oxygen species-driven pro-inflammatory phenotype in these MΦs.^200^ The tricarboxylic acid (TCA) cycle serves as a fundamental metabolic pathway within the mitochondria, mediating the oxidation of acetyl-CoA sourced from carbohydrates, lipids, and proteins to yield energy and key metabolic intermediates. Through this process, the TCA cycle supports oxidative phosphorylation for ATP synthesis. Beyond its role in energy production, the TCA cycle is integral to the regulation of MΦ metabolism and immune function. IRG1 is an enzyme induced by inflammation in MΦs that catalyses the conversion of the TCA cycle intermediate cis-aconitate into itaconate, thereby connecting cellular metabolism to both antimicrobial and anti-inflammatory responses. IRG1 expression has been documented in TAMs within both human and murine tumours. Importantly, deleting IRG1 in mice resulted in reduced tumour growth and improved responses to anti-PD-(L)1 immunotherapy.^201^

A recent study investigated therapeutic intervention in glioblastoma by inhibiting TAM-derived lactate transport or lactylation. When combined with immune checkpoint inhibitors, this strategy improved outcomes in immunocompetent xenograft models.^202^ Alternatively, glioblastoma-supportive macrophages (GSMs) were repolarized into anti-tumour MΦs through an intracavitary sprayable nanoregulator-encased hydrogel system that modulates cholesterol metabolism in GSMs.^203^ The targeting of the glutamine pathway has demonstrated considerable potential as a strategy for inhibiting metastatic progression and improving patient survival. Specifically, pharmacological inhibition of glutamine synthetase (GS) activity in MΦs, as well as genetic deletion of GS in murine models, induces a pro-inflammatory phenotype in these cells. This promotes the recruitment of cytotoxic T cells within the tumour microenvironment (TME) and suppresses metastasis.^204^ Given that arginine catabolism plays a significant role in mediating immunosuppression within the TME, multiple strategies have been developed to target this pathway with the aim of enhancing anti-cancer immunity. Several arginase-1 (Arg-1) inhibitors, such as CB-1158, have demonstrated efficacy in various murine cancer models—including melanoma, glioma, as well as breast and colorectal cancers.^205–207^ At present, a variety of clinical trials are underway to assess how targeting various metabolic pathways in MΦs might treat solid tumours. These include investigations into the glutamine metabolism pathway (NCT03944902, NCT02861300) and the OXPHOS pathway (NCT03272256).

## Setting the stage: inflammation-signaling of macrophages shaped by the tumor microenvironment (TME)

Whereas the previous chapter introduced strategies to externally modulate MΦ metabolism, it is equally important to consider how the local environment within tissues shapes MΦ behavior. In healthy tissue, MΦ s are guided by homeostatic signals, but under pathological conditions, including cancer, signaling cues can redirect their functions. The tumor microenvironment (TME), in particular, exploits the high plasticity of MΦs to promote tumor progression and therapy resistance. The TME is a complex-structured and dynamic network composed of cancer cells, extra cellular matrix components, tumor surrounding stromal cells, immune cells, mesenchymal stem cells and cancer-associated fibroblasts (CAFs).^208^ Among the most abundant immune cell populations in the TME are TAMs, which are initially monocytes that have been recruited to the tumor site by chemokines like CCL2 (via CCR2) and CXCL2 (via CXCR4). Once within the TME, monocytes are exposed to a range of tumor-derived factors such as IL-4, IL-10 and TGF-β, differentiating them towards an M2-like, pro-tumoral MΦ phenotype.^209^ Tumor cells play a central role by secreting colony-stimulating factor 1 (CSF-1) that activates downstream pathways including PI3K/AKT and MAPK/ERK, promoting TAM expansion and survival. Furthermore, regulatory T cells (Tregs)^210^ and myeloid-derived suppressor cells (MDSCs)^211^ enhance immunosuppression via secretion of TGF-ß, enforcing an anti-inflammatory and tissue-remodeling TAM phenotype. They also secrete IL-10, suppressing pro-inflammatory responses and further dampening antigen presentation. Once polarized, TAMs also contribute to the immunosuppression themselves, by upregulating the expression of immune checkpoint ligands such as PD-L1 and CD80, which bind to PD-1 and CTLA-4 on cytotoxic T lymphocytes (CTLs) and natural killer (NK) cells, leading to T cell exhaustion and functional anergy.^212,213^ Hypoxia is a common feature of the TME, resulting from rapid tumor growth outpacing its blood supply.^214^ It can further influence TAM function through stabilization of hypoxia-inducible factors (HIFs) under low oxygen conditions, altering MΦ behavior, thus shifting MΦs toward glycolysis and reinforcing their pro-tumoral role.^215^ Moreover, this promotes the induction of angiogenesis through secretion of VEGF and matrix metalloproteinases (MMPs).^216^ Furthermore, tumor-derived lactate can also drive the polarization of MΦs toward the M2 phenotype by activating the ERK and STAT3 signaling pathways.^217^ In addition to lactate-mediated effects, the acidic TME itself is sensed by MΦs through the proton-sensing G protein–coupled receptor GPR65, which activates the cAMP/PKA signaling cascade to promote an immunosuppressive, M2-like phenotype.^218^ This mechanism highlights that acidosis functions not merely as a metabolic by-product but as an active immunomodulatory signal within the TME. The growing recognition of GPR65-dependent MΦ polarization has spurred translational interest, exemplified by the initiation of the first phase 1/2 clinical trial evaluating a small-molecule GPR65 inhibitor (NCT06634849). Together, these findings underscore the multifaceted ways in which tumor-derived metabolic and acidic cues shape MΦ function and sustain tumor-promoting inflammation. Additionally, crosstalk between MΦs and tumor-associated stromal components generates complex feedback loops that finetune TAM polarization and activity. For instance, TAM and cancer-associated fibroblast (CAF) engagement can lead to enhanced ECM remodeling, creating a fibrotic and immunosuppressive TME.^219^ TGF-β, secreted by both cell types, drives CAF activation and ECM stiffening via the YAP/TAZ pathway, forming a physical barrier that limits immune cell infiltration.^220,221^ TAM-derived osteopontin (SPP1) and CAF-secreted CXCL12 further promote fibrosis and recruit Tregs and MDSCs through the CXCR4 receptor. This crosstalk of TAMs and the TME underscores the dynamic and adaptable nature of MΦs in cancer and highlights multiple potential therapeutic targets. Therefore, understanding these interactions and the underlying signaling pathways is crucial for developing strategies to modulate the TME and enhance anti-tumor immunity.

## Reprogramming the signaling of macrophages as a precision tool in anti-cancer therapies

**Fig. 2:**
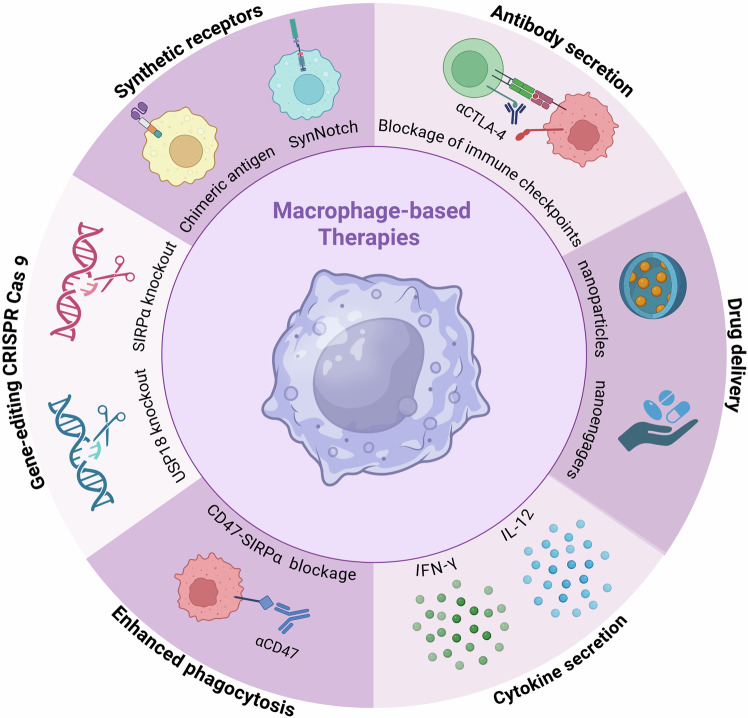
Schematic representation of engineered macrophages and their diverse therapeutic applications. Engineered MΦs can be modified via numerous strategies including the incorporation of synthetic receptors (e.g., chimeric antigen receptors, SynNotch), CRISPR/Cas9 gene editing (e.g., USP18 and SIRPα knockouts), and enhanced phagocytosis via CD47–SIRPα blockade. Additional functions include cytokine secretion (e.g., IFN-γ, IL-12), drug delivery using nanoparticles or nanoengagers, and antibody secretion to block immune checkpoints (e.g., αCTLA-4). These modifications collectively enhance MΦ efficacy in immunotherapy and disease treatment

The recent knowledge about MΦ plasticity and their interaction with and within the TME, has paved the way for harnessing MΦ function and to redirect them into an activation stage which is directed against the tumor. This has resulted in a powerful class of immunotherapy, particularly effective in solid tumors and inflammatory diseases. Despite this progress, therapeutic outcomes using naïve MΦs–those without pre-treatment or genetic modification—have remained limited. For example, although naïve non-polarized M0 MΦs initially showed anti-tumor activity against pancreatic ductal adenocarcinoma via TNF secretion, this effect diminished over time due to a forced polarization towards a pro-tumoral M2 phenotype, leading to a decline of cytotoxic anti-tumor efficacy.^222^ This data indicates the critical need to maintain or re-induce an anti-tumoral phenotype for sustained therapeutic benefits. To counteract the tumor-induced inhibitory signals, genetic engineering has been employed to create MΦs that resist such reprogramming strategies (Fig. 2). One important target is the CD47-SIRPα axis: CD47, often overexpressed on tumor cells, acts as a “don´t eat me” signal by binding to the inhibitory Signal Regulatory Protein α (SIRPα) receptor on MΦs.^223^ This interaction suppresses phagocytic signaling via SHP1/2-mediated pathways, reducing immune clearance of tumor cells. To further neutralize this inhibitory checkpoint, several therapeutic strategies have been developed. These include antibody-mediated CD47 blockade and genome-editing tools to disrupt SIRPα expression in MΦs such as via CRISPR/Cas9-based knockout techniques. For instance, SIRPα knockout MΦs derived from induced pluripotent stem cells (iPSCs) showed resistance to CD47-mediated inhibition and an enhanced antibody-dependent phagocytic and killing capacity across various tumor types including WM266-4 (melanoma), SKOV-3 (ovarian cancer), and MCF7 (breast cancer).^224,225^ Additionally, CRISPR-Cas9 has been used to modify intracellular signaling regulators such as USP18 (Ubiquitin Specific Peptidase 18), a suppressor of type I interferon (IFN) pathways. Knocking out USP18 in iPSC-derived MΦs (iMacs) enhanced the JAK/STAT signaling, further boosting the response to type I IFN, leading to a more robust antiviral response and reduced HIV-1 replication by upregulating antigen presentation machinery and inflammatory mediators.^226^ Beyond intrinsic modifications, strategies to disrupt the immunosuppressive TME include targeting the TAM–CAF–ECM network. One approach uses TGF-β inhibitors—currently being tested in combination with immune checkpoint inhibitors—to reduce fibrosis and promote T cell infiltration.^227^ Similarly, CXCR4 antagonists have been developed to block the immunosuppressive CXCL12/CXCR4 axis, facilitating effector T cell access to tumor sites.^228^ Another promising avenue is the enzymatic degradation of ECM components, e.g. through hyaluronidase, which decreases matrix stiffness and improves both drug delivery and immune cell penetration.^229^ Alternatively, MΦs can be engineered to secrete immunostimulatory cytokines or antibodies, thereby reshaping the immunosuppressive TME by recruiting and activating cytotoxic T and NK cells and upregulating antigen presentation pathways. This approach has been used in mouse hematopoietic stem and progenitor cells (HSPCs), which were differentiated into IL-12-secreting myeloid cells, reshaping the immunosuppressive TME and improving survival in a rhabdomyosarcoma model.^230^ In related work, human IL-12-producing iMacs boosted cytotoxic CD8^+^ T cell responses and enhanced tumor cell killing in a xenograft mouse model of human lung cancer.^231^ Further supporting this concept, engineered murine MΦs expressing IFN-y restored alveolar immunity, increased MHC II expression, and improved phagocytosis in immunodeficient mice.^232^ To strengthen adaptive immune responses and mitigate cytotoxic T cell exhaustion, a recent study has modified human monocyte-derived MΦs to locally deliver checkpoint inhibitors like anti-CTLA-4.^233^ Similar targeted approaches were also used in the context of chronic inflammatory diseases, where engineered murine iMacs produced self-regulated and feedback-controlled soluble TNF receptor 1 (sTNFR1) in response to TNF-α levels.^234^ In a related study, human monocyte-derived MΦs were modified to secrete anti-TNF antibodies to treat rheumatoid arthritis (RA).^233^ Beyond acting as effectors, MΦs are also being explored as delivery vehicles due to their natural migratory and phagocytic properties, which they can use for homing to disease sites and releasing therapeutics in a controlled manner. When equipped with targeting ligands—such as anti-HER2 affibodies—and loaded with doxorubicin-carrying nanoparticles, genetically-engineered MΦs (GEMs) demonstrated targeted delivery and accumulation in HER2^+^ tumors, enhancing chemotherapy outcomes through synergistic effects.^235^ To address resistance in immunologically “cold” tumors like pancreatic cancer, recent studies developed GEM membrane-based nanoengagers. These displayed bispecific ligands targeting Claudin 18.2 on pancreatic cancer cells and CD40 on MΦs, leading to MΦ activation, NF-κB signaling and increased pro-inflammatory cytokine release and antigen presentation, demonstrating effective reprogramming of the TME, further improving cytotoxic T cell responses.^236^ To fine-tune antigen-specific immune responses, MΦs are being equipped with synthetic receptors like chimeric antigen- (CAR) or synNotch receptors, combining an extracellular recognition domain with a customizable transcriptional response module, enabling a precise and controlled gene expression. So far, synNotch-expressing MΦs were used in the context of non-invasive cancer diagnostics, with synNotch-triggered human chorionic gonadotropin (HCGB5) secretion upon binding to tumor cells, offering a promising platform for real-time disease monitoring.^237^ To further investigate the interactions between TAMs and cancer cells in the TME, another study used αCD206-synNotch-expressing cancer cells to detect TAMs and thus, acting as reporter cells upon binding.^238^ In efforts to reverse the immunosuppressive functions of recruited and reprogrammed TAMs and restore anti-tumor activity, several in vivo MΦ-targeted therapies have been developed. CSF-1R inhibitors, for instance, block the CSF-1/CSF-1R axis, thereby reducing M2-like MΦs through inhibition of PI3K/AKT and ERK signaling.^239^ To further promote pro-inflammatory activation, TLR agonists like R848 are used to stimulate TLR7/8, triggering a MyD88-dependent NF-κB and IRF7 signaling. This shifts MΦs towards an M1 phenotype, boosting pro-inflammatory cytokine release (e.g., TNF-α, IL-12) and enhancing anti-tumor immunity. In addition, small molecules and monoclonal antibodies are also being used to reprogram M2-like TAMs by inhibiting STAT3 or activating JAK/STAT1 pathways, restoring antigen presentation and costimulatory molecule expression.^240^ These strategies are often combined with immune checkpoint inhibitors, chemotherapy, or radiotherapy to amplify immune responses, and enhance T cell infiltration, overall improving tumor sensitivity to treatment.^241^ In parallel, a rapidly emerging strategy is in situ MΦ engineering, which seeks to reprogram TAMs directly within the TME without ex vivo manipulation. By harnessing the intrinsic abundance, phagocytic capacity, and phenotypic plasticity of intratumoral MΦs, this strategy enables direct functional modulation while preserving the TME context typically compromised by ex vivo approaches. To this end, different approaches are currently under investigation, highlighting advanced lipid nanoparticle (LNP) systems, polymeric carriers, as well as viral or non-viral vectors that deliver mRNA, siRNA, or CRISPR components to MΦs in order to achieve a pro-inflammatory gene expression signature or to silence immunosuppressive mediators within MΦs.^242,243^ These delivery platforms are often engineered to exploit MΦ-specific uptake pathways, such as scavenger receptors, Fc receptors, or mannose receptors, thereby improving selectivity toward myeloid cells while limiting off-target effects in non-immune tissues. One notable approach utilizes gold nanoparticle-based nanozymes that release TLR7/8 agonists within MΦs, driving NF-κB activation and conversion of M2-like TAMs into M1-like effector cells with enhanced phagocytic and antitumor activity. These nanozymes use the catalytic activity of gold nanoparticles to alter intracellular redox states, synergize with TLR signaling, and enhance inflammation, resulting in increased cytokine production, improved antigen uptake, stronger CD8⁺ T cell priming, and tumor regression in preclinical models.^244^ Similarly, LNP-based systems have attracted substantial attention due to their clinical validation in mRNA vaccines and their modularity in terms of lipid composition, size, and surface functionalization. By adjusting ionizable lipids, and targeting ligands, LNPs can be optimized for preferential uptake by MΦs residing in tumors.^245^ LNPs delivering mRNA encoding pro-inflammatory transcription factors, costimulatory molecules, or cytokines (e.g., IL-12) have been shown in preclinical models to induce durable pro-inflammatory polarization of TAMs and increased recruitment of cytotoxic lymphocytes, resulting in overall enhanced anti-tumor responses.^246^ In addition, LNPs loaded with siRNA targeting inhibitory regulators have been shown to selectively silence TAMs in situ, restoring immune surveillance and improving responses to checkpoint blockade.^243,247^ For example, siRNA-mediated knockdown of PD-L1, STAT3,^248,249^ or C/EBPβ^250^ in TAMs shifted MΦ polarization toward an immunostimulatory phenotype with enhanced antitumor efficacy and sensitized tumors to anti-PD-1 or anti-CTLA-4 therapy. Furthermore, hydrogel or scaffold-based depots capable of releasing MΦ-modulating cytokines or RNA cargos locally provide spatially controlled reprogramming while minimizing systemic exposure.^251,252^ Injectable hydrogels releasing TLR7/8 agonists^253^ or GM-CSF^254^ have been shown to reprogram infiltrating MΦs, promote tertiary lymphoid structure formation,^255^ and enhance antitumor immune memory in murine models, further synergizing with anti-PD-1 therapy. In addition to nanoparticle-based delivery systems, an alternative system is the use of viral and non-viral vectors, being explored for in situ MΦ engineering. Lentiviral vectors (LVs) can efficiently transduce both dividing and non-dividing cells and enable stable genomic integration, allowing sustained expression of MΦ-reprogramming factors.^256,257^ In tumor models, genetic or viral engineering approaches involving lentiviral delivery of IL-12,^213^ NF-κB pathway modulation, or inhibition of STAT3 signaling have been shown to reprogram TAMs toward inflammatory phenotypes, enhance antigen presentation, and promote T cell–mediated tumor control.^258–260^ MΦ selectivity can be further enhanced through envelope pseudotyping or incorporation of myeloid-targeting ligands. Furthermore, Adeno-associated viral (AAV) vectors represent a transient viral strategy with a favorable safety profile and predominantly episomal persistence.^261^ Although conventional AAV serotypes show limited MΦ tropism, capsid-engineered AAV variants with enhanced myeloid transduction have recently been described.^262^ These vectors have been used to deliver immunomodulatory transgenes or CRISPR/Cas9 components to myeloid cells in vivo,^263^ enabling transient but effective modulation of MΦ function within tumors. AAV-mediated expression of IL-12^264^ or IFN-β^265^ in the TME has been shown to enhance cytotoxic lymphocyte infiltration and suppress tumor growth in murine cancer models.

Despite these advances, viral vector–based approaches face challenges including pre-existing immunity, limited repeat dosing, insertional mutagenesis risk (for integrating vectors), and manufacturing complexity. To address these issues, non-integrating lentiviral vectors, self-limiting expression cassettes, and hybrid viral–nanoparticle systems are currently under development.^256,266,267^ In parallel, non-viral alternatives such as minicircle DNA and self-amplifying RNA vectors are gaining interest as they can recapitulate many of the expression advantages of viral systems while minimizing immunogenicity and genomic risk. Together, these in situ engineering approaches could offer flexible means to re-educate MΦs and reshape the tumor immune landscape. Whether the data will be clinically translatable has yet to be shown and will critically depend on the ability to target sufficient cells and the dose-limiting toxicities of LNPs and other delivery vehicles in these settings. Beyond genetic and pharmacologic approaches, metabolic regulators are emerging as powerful tools to modulate TAM function. Recently described chemosensors, for example, such as olfactory and vomeronasal receptors, have been identified as key regulators of TAM polarization, promoting a tumor-supportive phenotype across multiple cancers. Targeting these receptors, for example by deleting OR51E2 or interfering with its ligand palmitic acid, can reprogram TAMs, reduce tumor growth, and enhance infiltration of tumor-reactive CD8⁺ T cells, highlighting chemosensors as potential immunotherapy targets.^268^ By controlling processes such as glycolysis, oxidative phosphorylation, and lipid metabolism, these strategies can fine-tune MΦ polarization and functional plasticity in a context-dependent manner. Using metabolically-responsive systems with nanoparticle- or biomaterial-based delivery platforms could further enhance specificity and efficacy, enabling spatially and temporally precise TAM reprogramming within the TME. Such approaches open new avenues for combining metabolic modulation with existing immunotherapies or GEM-based strategies, potentially amplifying anti-tumor immune responses while limiting systemic toxicity. Altogether, these pharmacologic approaches complement GEM-based therapies, offering a versatile strategy to remodel the TME and improve therapeutic outcomes. In summary, MΦs are highly responsive to diverse signaling pathways, making them ideal candidates for therapeutic engineering. Their inherent plasticity enables both suppression of pro-tumoral signals and enhancement of immune-stimulating functions. In conclusion, GEMs represent a promising platform for next-generation cell-based immunotherapies in oncology and targeted drug delivery.

## Synthetic biology and concepts to change the fate of macrophages: benefits compared to CAR-T and CAR-NK therapies

**Fig. 3:**
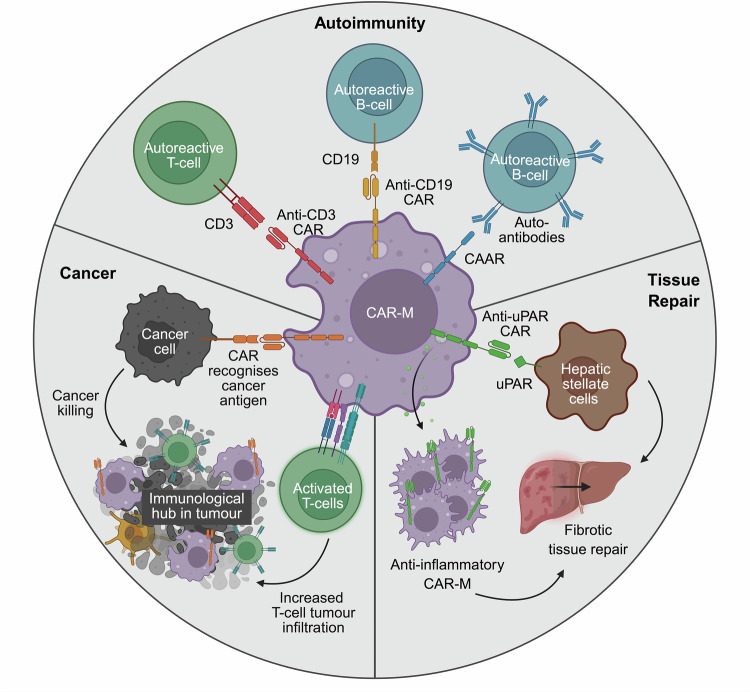
Use of CAR-Macrophage application in various diseases and to direct their function either to pro- or anti-inflammatory signaling. *Cancer*: Enhancing phagocytosis and interacting with T cells, forming an immunological hub in the tumour (pro-inflammation). *Autoimmunity*: Eradicating auto-reactive CD19^+^ B and CD3^+^ T cells (pro-inflammation). *Tissue repair*: Resolving fibrosis in the liver (anti-inflammation)

While the plasticity of MΦ has been an attractive target to modulate their anti-tumoral fate in vivo, adaptive MΦ therapy emerged as a promising approach in the context of solid tumors due to its infiltration capacity and lower concern for graft-versus-host disease (GvHD) compared to other cell-based immunotherapies. The autologous transfer of IFN-γ- and LPS-stimulated M1 MΦs to solid tumor patients did not prove its efficacy, although adverse effect was low grade.^269^ Using the knowledge from the TME and TAMs as well as clinical data from T and Natural Killer (NK) cell immunotherapies, the use of CAR technology for MΦs is hence a pivotal advancement in adaptive MΦ therapy. The structure of a CAR is composed of a single chain variable fragment (scFv), hinge, transmembrane domain, and an intracellular signal domain. Antigen binding via the scFv facilitates dimer formation of CARs, resulting in intracellular signal domain activation. The first and second generations of CARs were designed for use in T cells, both consisting of a CD8 hinge and transmembrane domain as well as a CD3ζ intracellular signaling domain. The CD3ζ domain is derived from the T-cell receptor (TCR)-CD3 complex and consists of three immunoreceptor tyrosine-based activation motifs (ITAMs) that are crucial to initiate signaling.^270^ In CAR-T cells, lymphocyte-specific protein tyrosine kinase (LCK) phosphorylates the ITAMs and recruits kinases such as the zeta chain-associated protein kinase of 70 kDa (ZAP-70) to further activate downstream signals. To improve clinical efficacy of CAR therapies, the second generation further attempts to improve the function and survival of CAR-cells by adding a single co-stimulatory domain, such as CD28 or 4-1BB, whereas third generation CARs used combinations of these domains. While initially conceptualized for T cells, the same CAR structure design was also applied for CAR-NK cells, as shown in a clinical trial for blood cancer.^271^ Perhaps due to the ability of CD3ζ to signal NKp30 and CD16, the same design can also activate NK cells’ anti-cancer ability.^272^ While both CAR-T and CAR-NK were used successfully for blood cancer, their efficacy is limited in solid tumors. In their stead, CAR-macrophages (CAR-Ms) could leverage their tumor-infiltrating capacity to allow targeting of solid tumors. Intriguingly, CD3ζ has also been widely adopted in CAR-M studies,^273^ including clinical trials of CAR-Ms,^274^ which will be discussed in a later section. A potential explanation lies in the shared ITAMs between CD3ζ and MΦ receptors like FcγR and Dectin-1, suggesting CD3ζ may mimic endogenous activation pathways to trigger innate immune responses.^275,276^ Despite similarities in using a CD3ζ intracellular domain, CAR-T, CAR-NK and CAR-M have distinct mechanisms of action and signaling pathways. When CAR-T cells recognise tumour antigens, they release perforin, granzymes, and pro-inflammatory cytokines such as IL-6, IL-10, and MCP-1 to induce pyroptosis. Yet, these molecules can cause severe cytokine release syndrome (CRS), which is one of the main adverse events reported in CAR-T cell clinical studies. Although CAR-NK cells can also secrete pro-inflammatory cytokines, they secrete a different set of cytokines that are not associated with CRS. Similarly, CAR-M can also secrete pro-inflammatory cytokines, but both CAR-NK and CAR-M have shorter lifespans in vivo compared to CAR-T cells. The limited persistence may affect efficacy to a certain extent, but on the other hand, it is beneficial in terms of lower long-term toxicity, thus, reducing adverse events during administration. In fact, CAR-M therapy demonstrated an excellent safety profile, with no grade 3-4 adverse events reported—a stark contrast to CAR-T cells.^277^ This safety advantage, shared with CAR-NK cell therapies,^271^ positions CAR-Ms as a compelling platform for further development. CAR-Ms can exhibit enhanced phagocytosis and inflammatory cytokine production when targeting specific antigens.^278,279^ Furthermore, the mechanism of CAR-M also relies on their superior tumour infiltration ability, caused by e.g. secretion of MMPs to degrade the tumour extracellular matrix, allowing higher infiltration of immune cells.^280^ Of note, CAR-Ms were applied to both hematological malignancies^281^ and solid tumors^278^ as well as other disease indications (Fig. 3). Their superior tumor infiltration capacity compared to CAR-T cells drove early clinical trials in solid cancers, with promising tumor reduction observed in HER2^high^ patients.^274^ These studies further suggest that CAR signaling can modulate MΦ chemotaxis, indicating the need to design CARs that align with the behaviour of MΦs. Current approaches to design CAR structures borrow heavily from T cell paradigms that modify co-stimulatory domains. The majority of CAR-T-, CAR-NK-, and CAR-M cells share a common modular signaling pathway, from scFv antigen-recognition to ITAM-based activation, co-stimulation motifs, and downstream functional pathways. Across all three lineages, CAR recognition often induces phosphorylation of ITAM domains that can recruit kinases (e.g. ZAP-70/Syk-family kinases^272^), and lead to activation of cascade pathways (e.g. PI3K-AKT, MAPK, and NF-κB^270^). However, the amplitude and downstream transcriptional outcomes are shaped by lineage-specific processes. Thus, the endogenous signaling architectures and effector programs of the different cell types should be taken into consideration. In T-cells, canonical costimulatory domains, including CD28 and 4-1BB, can drive clonal expansion and effector cytokine production, but also predispose to T-cell exhaustion, CRS and neurotoxicity. In CAR-NK cell design, signaling adapters like DAP12^282^ and 2B4^283^ are involved in activating NK cells and can be a better alternative to CD3ζ. Moreover, NK cells can kill tumour cells non-specifically due to their lack of MHC restriction, and CAR-NK can remain partly CAR-independent due to its natural cytotoxic behaviour.^284^ However, the limited persistence of NK cells in vivo restricts efficacy, and the immunosuppressive TME may impair the functions of CAR-NK cells. On the other hand, MΦ tumor-killing efficacy may be more dominated by phagocytic clearance and TME remodeling. As a result, CAR-M constructs typically repurpose CD3ζ and MΦ receptors like FcγR, along with TLR intracellular modules to induce phagocytosis and pro-inflammatory phenotypes in the TME. Therefore, MΦ-specific signaling domains or combinatorial motifs, such as phagocytosis-promoting signals like MerTK, may be necessary to fully exploit their therapeutic potential.^285^ Although the persistence of CAR-M cells in vivo can vary depending on MΦ phenotype, their ability to remodel the TME and recruit other immune cells presents an advantage over CAR-T- and CAR-NK cells. Table 1 summarises the key differences between CAR-T, CAR-NK and CAR-M therapies.

**Table 1:** Key differences between CAR-T, CAR-NK and CAR-M therapies

Features	CAR-T	CAR-NK	CAR-M
Tumour infiltration	Limited infiltration in solid tumor due to immunosuppressive TME	Moderate infiltration in solid tumors	High infiltration in solid tumors, can penetrate tumor regions and remodel TME^278^
In vivo persistence	Relatively long-lived with proliferative ability and potential memory T cell development	Generally shorter lifespan with limited proliferative potential^383,384^	Moderate persistence, depending on MΦ phenotype
Safety	Relatively high risk of GvHD, CRS and neurotoxicity^385^	Low to no risk of GvHD, CRS and neurotoxicity^385^	Current studies indicate favorable safety profile with no high-grade adverse events
Manufacturing complexity	Complex, costly, and time-consuming for autologous manufacturing of individual patients	Less complex and “off-the-shelf” products are possible due to reduced risk of GvHD and lower alloreactivity	Emerging technologies and methods for large-scale manufacturing are under development

While CAR-M refinement remains critical for cancer applications, their role in tissue homeostasis suggests broader therapeutic potential, such as in fibrotic tissue repair,^286^ cardiovascular,^115^ neurological^287^ and autoimmune diseases. In fact, engineered M2-like MΦs have shown promise in resolving fibrotic disease,^163^ and direct targeting of pathogenic B-cells, such as CD19^+^ B cell depletion in systemic lupus erythematosus (SLE),^288^ or autoantibody-specific chimeric autoantibody receptor (CAAR) approaches,^289^ could reset immune dysregulation. Similar to CAR-T_regs_ being explored for transplant tolerance,^290^ identifying key regulators of MΦs can allow CAR-Ms to be programmed for activation of immunosuppressive genetic pathways for tissue repair and autoimmune disease. Developing CARs that skew MΦs toward M2-like phenotypes could be an effective approach for ameliorating inflammatory tissue damage. For example, Cao et al. used an anti-TNF-CAR with an IL-4 signaling domain to induce anti-inflammatory programs.^291^ Here, the recognition of inflammatory ligand TNF switched CAR-Ms to an inflammation-suppressive phenotype and ameliorates renal damage in murine models.

To further fine-tune CAR-M functions, precision control systems with humanized notch-based receptors (e.g., Synthetic Notch (SynNotch) and synthetic intramembrane proteolysis receptor (SNIPR)^292^ can enable antigen-specific activation of genetic programs. Integrating immune-regulatory domains, such as anti-inflammatory cytokines or checkpoint blockers, into these systems could enhance CAR-M activity. Additionally, lessons learnt from CAR-T function optimization could inspire analogous CAR-M improvements.^48^ In a particular study, T cell leukemia mutations were screened and repurposed to enhance CAR-T cell function. One of the mutations, a CARD11–PIK3R3 fusion protein, was identified in an in vivo screening and shown to significantly enhance CAR-T cell function in animal models. This study demonstrates the importance of in vivo screenings and further encourages similar screening projects in the context of CAR-Ms. For instance, modulating epigenetic remodelers and conducting base-editor screens may be a good opportunity to identify novel regulators for enhancing CAR-M function for tissue repair and resolving autoimmunity. Therefore, to achieve new milestones for CAR-M optimization, functional screenings in vivo are crucial.

Recent technological advances in CAR-T engineering also demonstrate new opportunities for CAR-M development. Emerging approaches like scFv-secreting CAR-T cells enable dual targeting—engaging tumor-associated antigens while neutralizing immune checkpoints such as PD-1 blockade.^293^ Similarly, CAR-T cells secreting bispecific T cell engagers (BiTE) have shown efficacy in glioblastoma (GBM) with heterogeneous antigen expression.^294^ These advances highlight the potential for CAR-Ms to secrete therapeutics locally, such as checkpoint inhibitors or cytokines, within poorly penetrable solid tumors. Secretion of immunostimulatory molecules can reshape the TME, upregulating antigen presentation pathways for recruiting and engaging other immune effector cells, such as cytotoxic T and NK cells.^258^ Along with the potential to reprogram the TAMs into pro-inflammatory phenotypes, CAR-Ms can synergize with other immune cells and consequently establish a more robust attack on the tumor cells. Building on this concept, another approach currently under investigation is the combination of CAR-Ms with CAR-T cells. In such a tandem, CAR-Ms may be used to overcome the immunosuppressive environment, paving the way for CAR T cells to carry out their specific anti-tumor-clearance capacity; in turn, CAR T-cell-derived signals further boost CAR-M activity, allowing both cell types to cooperate more effectively. An additional concept was shown by Jing et al., demonstrating a modular LNP-based platform that delivers circular RNAs encoding an anti-CA9 CAR together with an IL-2R-TLR4 chimeric receptor, supported by a hydrogel providing local IL-2, to sustain MΦ M1 polarization and enhance tumor phagocytosis. This coordinated system simultaneously promoted antigen presentation and robust CD4⁺ and CD8⁺ T-cell recruitment, yielding a synergistic innate and adaptive antitumor response capable of suppressing tumor growth and recurrence in RCC models.^295–297^ Similarly, an allogeneic, off-the-shelf CAR-M approach has been developed targeting the prostate stem cell antigen (PSCA) and expressing membrane-bound IL-15, which stimulates T-cell activation as well as NK-cell proliferation and cytotoxicity. By engaging multiple arms of the immune system, this strategy enhances effector cell infiltration, overrides immunosuppressive TAMs, and further strengthens the synergistic antitumor activity of CAR-Ms in solid tumors.^298,299^ By leveraging their innate tumor-infiltrating ability or generating them in vivo for sustained activity, CAR-Ms can complement therapies like PD-1 blockade, which in turn enhances CAR-M efficacy by boosting tumor-infiltrating lymphocytes (TILs).^258^ Hence, CAR-M combination therapy approaches can convert immunologically “cold” tumors into “hot” tumors,^258^ increasing the efficacy of the treatment. This positions CAR-Ms as a bridge between innate and adaptive immunity, with potential applications in neoadjuvant immunotherapy to prime antitumor responses pre-surgery. Thus, further advances in CAR-M engineering will undoubtedly lead to a broader range of therapeutic applications.

### From signaling cascades to novel CAR designs and optimization strategies for myeloid cells

**Fig. 4:**
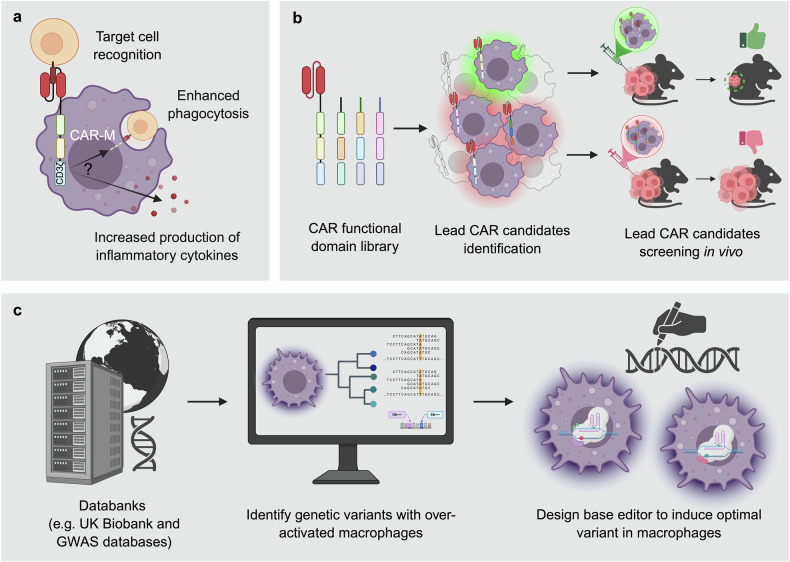
Next-generation CAR design for macrophages. **a** CAR-Ms with CD3ζ intracellular signaling domain. The exact downstream signals of CD3ζ signaling are unknown, but it can enhance phagocytosis and increase the production of pro-inflammatory cytokines. **b** CAR-pooled screening can be used to identify lead CAR candidates in vivo and screen for functionally enhanced CAR-Ms with particular CAR structural domains. **c** Utilising existing databases such as the UK Biobank or GWAS profiling can help identify genetic variants associated with over-activated MΦs. This aids the development of base-editing methods to induce the optimal variant in MΦs and facilitates the creation of better CAR designs for specific functions

CAR design for MΦs requires strategic and precise optimization, given the broad functional repertoire of these cells. Early CAR designs for MΦs, such as the FcγR-based construct,^285^ demonstrated enhanced phagocytosis without relying on CD3ζ (Fig. 4a). Subsequent innovations, like integrating TLR4 intracellular toll/IL-1R (TIR) domain into CD3ζ-based CARs,^300^ further boosted inflammatory cytokine production. However, high-throughput screening of signaling domains—akin to advancements in CAR-T cells^301^—remains critical for identifying novel configurations (Fig. 4b). A key challenge is balancing CAR complexity with production, as oversized structures hinder transduction efficiency, particularly in primary MΦs, which are resistant to lentiviral/retroviral vectors and electroporation.^302,303^ This reliance on adenoviral delivery, which is less accessible to many labs, underscores the need for new engineering approaches and designs. Genome-wide CRISPR screening could identify regulators to enhance CAR-M efficacy. For example, in vivo CRISPR screens in CAR-NK cells revealed surface proteins that improve tumor retention^304^—a strategy that could also be beneficial in CAR-M research. Parallel efforts to boost MΦ functionalities, such as phagocytosis, include Rac hyperactivation mutants, inspired by Drosophila ovary biology,^305^ which enhance tumor engulfment. Also, recent work targeting the “don’t eat me” signal SIRPα identified Vav1 as a downstream effector, showing that hyperactivating Vav1 amplifies phagocytosis.^306^ Apart from improving MΦ functionalities, MΦs can also synergize with other immune cells. Central inflammatory mediators like SYK and MyD88 (downstream of TLR pathways) drive chemokine production and T cell recruitment, bridging innate and adaptive immunity. Integrating SYK and MyD88 domains into CAR designs represents a promising frontier.

Beyond de novo screenings, insights could arise from evolutionary adaptations. For instance, information derived from the UK Biobank or GWAS databases could offer potentially important variants that regulate MΦ functions (Fig. 4c), which may be analogous to the hyperactivating mutations identified in CAR-T studies.^307^ Similarly, STAT1 gain-of-function mutations may offer potential targets inspired by primary immunodeficiency patients whose MΦs are aberrantly overactivated.^308^ To pinpoint new variants, base-editor screening of signaling motifs may also identify modulators for CAR-M optimization. Again, drawing inspiration from CAR-T, cell signal can be affected by several parallel pathways. Recent studies suggested that TCR and 4-1BB tonic signaling can be leveraged to enhance CAR-T cell function.^309,310^ Interestingly, the TCR study implemented a machine-learning model to predict the strength of both TCR and CAR signaling and identified their optimal balance to maximize CAR-T cell function. Artificial intelligence, machine learning and advancements in computational biology methods will undoubtedly be a new frontier to guide CAR-M engineering more rationally. Functional multiplexing via screening, such as high-throughput CAR domain screening, based on CAR-T cell pool strategies, could identify optimal signaling combinations for MΦs. Likewise, integrative omics enables a deeper understanding of the underlying mechanisms and potential combinatory targets controlling CAR-M phenotypes. Such integrated datasets may also reveal metabolic changes associated with CAR-M polarization states, guiding metabolic reprogramming strategies to enhance CAR-M therapeutic efficacy. For functional analysis, single-cell RNA sequencing (scRNA-seq) can map functional clusters such as phagocytosis, cytotoxicity and antigen presentation to specific CAR signaling domains, enabling rational design. These domains can further be elucidated by structural biology-driven approaches. For example, utilizing cryo-electron tomography (Cryo-ET) on top-performing CAR-M constructs can visualize and refine CAR structural design. Along this line, spatial orientation, such as rotational positioning of the N-terminal domain, has been shown to affect antigen engagement and signaling crosstalk.^311^ However, current key engineering priorities are hinge-domain optimization for CAR protein trafficking to the cellular membrane and structural modifications (e.g. hinge length, flexibility) that will significantly impact in vivo efficacy. Eventually, predictive modeling using data derived from these computational and structural frameworks may be able to simulate CAR-M dynamics, optimize CAR design, dosing, and patient-specific treatment strategies by anticipating therapy responses and patterns.

### Current challenges and future directions in CAR-M Therapy

**Fig. 5:**
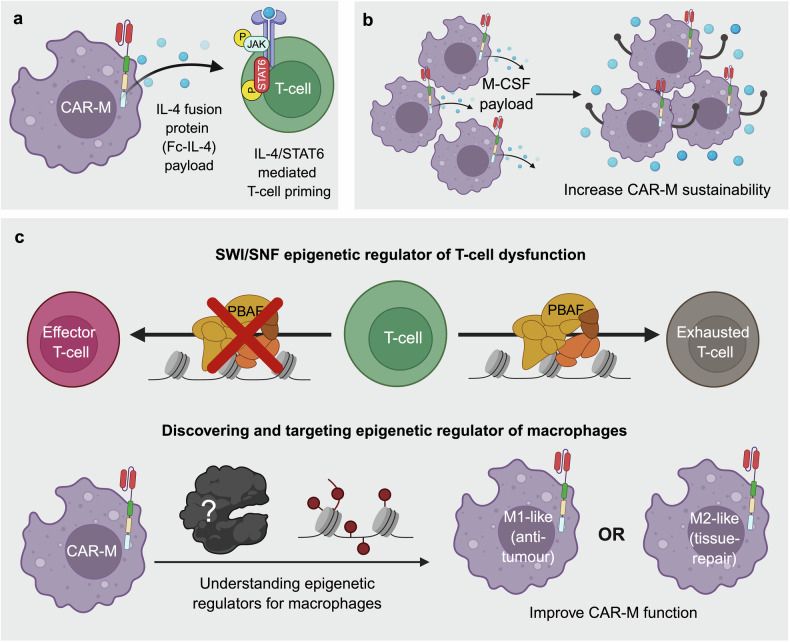
Future approaches to improve CAR-Macrophages. **a** Designing CAR-Ms to employ Th2 immunity by secreting Fc-IL-4 payload, which can subsequently induce T cell priming by activating the STAT6 pathway. **b** Increasing the sustainability of CAR-Ms in vivo by secreting M-CSF payload as a growth factor support, allowing long-term survival of CAR-Ms. **c** The SWI/SNF complex PBAF is a key epigenetic regulator of T cell dysfunction and can skew T cells towards a terminally exhausted phenotype. Understanding the corresponding mechanisms of epigenetic regulators in MΦss may help discover new targets to improve CAR-M function and skew CAR-Ms towards a beneficial phenotype

One of the main challenges in applying CAR-Ms in anti-tumor therapies is to avoid M2-like re-polarization within the TME. Strategies to sustain pro-inflammatory activity include payload engineering. Co-expressing Th1-polarizing cytokines (e.g., IFN-γ, IL-12) or dominant-negative receptors for M2-inducing signals (e.g., IL-4Rα truncation or modified TGFBRII) are promising attempts. Emerging evidence from CAR-T cell studies suggests that revisiting Th2 immunity—particularly IL-4/Th2 pathways—may enhance persistence and efficacy in patients.^312,313^ For CAR-Ms, Th2 signals governed by IL-4/STAT6 could synergize in T cell priming, though this requires careful balancing to avoid pro-tumor effects. One solution could be base editor screening of the ideal STAT variant that balances Th2 immunity with avoiding tumorigenic MΦs (Fig. 5a). The second challenge is limited CAR-M persistence. Growth factor support by M-CSF supplementation extends survival in preclinical models.^314^ In vivo screening inspired by CAR-NK screens identifying pro-survival surface proteins and knocking out CALHM2 could be adapted (Fig. 5b).^304^ A hybrid approach combining genome-wide in vivo screening for tumor-specific factors with scalable in vitro assays such as CRISPR screens under TME-mimetic conditions may prioritize hits for validation. The third challenge is organ-specific trafficking. Current systemic delivery leads to the aggregation of CAR-Ms in the lungs and liver. Solutions include targeted homing by antibody-coated CAR-Ms or engineering chemokine receptors, like CXCR4 for leukemia in bone marrow. In vivo reprogramming with mRNA-LNP delivery of CAR constructs to resident MΦs, such as in hepatocellular carcinoma, is another innovative approach.^315^ A recent comparative analysis of CAR-T-, CAR-NK cells, and CAR-Ms in glioblastoma found that payload design (e.g., IL-15/IL-21 priming) outweighed immune cell type in determining infiltration and efficacy.^284^ This underscores the need for modular cytokine circuits by integrating sensors for hypoxia or tumor-derived ATP to trigger localized cytokine release. Since microenvironment signals often influence MΦs through metabolic rewiring, manipulating MΦ metabolism can aid in enhancing therapeutic efficacy (as previously reviewed^316^). As such, ODN1826, a Class B CpG oligonucleotid for example, has been shown to activate TLR9 while changing the metabolic profile of MΦs at the same time, which enables cells to phagocytose CD47^+^ cancer cells.^317^ In a different approach targeting the itaconic acid pathway, depletion of ACOD1 in iPSC-derived MΦs could show a strong anti-tumor effect.^318^ The use of dual-armored CARs by combining checkpoint blockades, such as PD-1 scFv secretion, with metabolic reprogramming, is promising.

Key innovations for next-generation CAR-Ms include dynamic polarization, which can be mediated and controlled by SynNotch-driven switches to toggle between M1 (anti-tumor) and M2 (tissue-repair) states post-infiltration. Advancements in computational-guided design with machine learning can aid the prediction of optimal CAR structures (Fig. 4c), including hinge domains affecting spatial antigen engagement, as shown in previous studies.^311^ Evolution-inspired engineering leverages mutations from hyperinflammatory disorders, for example, STAT1 gain-of-function to enhance CAR-M resilience.^308^ Discovering epigenetic modifiers to sustain CAR-M function is crucial. Preceding studies in CAR-T cells give insight on rewiring epigenetic mechanisms as a beneficial approach. SWI/SNF complex PBAF maintains terminally-exhausted T cells, offering it as an interesting target to be tested in MΦs (Fig. 5c).^319^ Blocking JAK can delay terminal exhaustion of T cells through dynamic epigenetic changes, whereas some patients with high basal levels of IFN-γ did not respond.^320^ This insinuates another significant challenge in advancing CAR-M therapies. Unlike CAR-T therapy, where predictive biomarkers have been established for outcomes and adverse effects,^321^ there is a lack of extensive data on such patient stratification and selection markers for CAR-M therapy. Recent works suggest potential biomarkers such as those related to TAM infiltration, TME modulation, and MΦ polarization states. However, more specific studies are needed to deepen our understanding of biomarker-driven patient stratification to tailor CAR-M therapy for different patients. With more clinical data available, addressing the cell-autonomous mechanisms for CAR-M responders vs non-responders will offer new strategies to engineer better and improved cells for the clinical translation of CAR-Ms into patients suffering from solid malignancies and beyond.

## From basic macrophage biology to clinical application of engineered macrophages

While various pre-clinical techniques have been investigated to shift the fate of MΦs to required pro or anti-inflammatory states, engineered MΦs have already entered the clinic. Recent clinical applications of engineered MΦs have sought to exploit their phenotypic plasticity by utilizing their anti-inflammatory properties in regenerative medicine^322–324^ or engineering pro-inflammatory MΦs for oncology.^274^ The broad landscape and history of MΦ-based therapies have been previously reviewed^325,326^; this chapter highlights recent therapeutic applications and the challenges and future directions of this therapeutic modality.

Early clinical adoption of ex vivo IFN-γ-stimulated autologous MΦs for solid tumors showed little to no clinical efficacy^327^, perhaps linked to the limited persistence of the pro-inflammatory phenotype in the TME. Klichinsky et al.^278^ showed in a pre-clinical study that transducing primary human MΦs with a first generation anti-HER2 CAR with a chimeric adenoviral vector Ad5f35^328^ led to a persistent pro-inflammatory phenotype with sustained, reproducible CAR expression in transduced cells, overcoming the natural resistance of MΦs to the insertion of genetic material. The design of the CAR was inspired by first-generation CAR constructs with a CD3ζ signal domain, which have also shown robust anti-tumor responses in hPSC-derived CAR-Ms.^329^ Similarly, murine experiments using SKOV3 injected i.v. or i.p. into NSGS mice showed human CAR-Ms led to a significant reduction in tumor burden compared to untransduced MΦs,^278^ subsequently replicated in a murine CAR-M system.^258^ Consequently, autologous-derived human CAR-Ms were tested in a Phase I basket trial for patients with demonstrated HER2^+^ tumor expression (NCT04660929).

The latest published interim analysis of this trial^274^ described 14 patients infused with autologous anti-HER2 CAR-Ms, where CD14^+^ monocytes were isolated, differentiated into MΦs and transduced with the anti-HER2 CAR. The pre-infusion assessment of manufactured products validated pre-clinical findings, namely the retained capacity of CAR-Ms to eradicate cancer cells in vitro, including SKOV3 and polarization of CAR-Ms to a pro-inflammatory state through transfection.^274^ The trial specified a maximum dose of 5 × 10^9 cells, although only median doses of between 1.61 × 10^9 and 2.10 × 10^9 cells were infused across two patient groups. The treatment was well tolerated; there was no detection of adverse events (AEs) often seen in CAR-T cell treatments such as grade 3 cytokine release syndrome (CRS). Notably, the omission of lymphodepleting chemotherapy also helped patients avoid AEs such as prolonged immunosuppression. Nevertheless, the monotherapy efficacy of CAR-Ms was limited, with a Best Overall Response of Stable Disease (SD) observed in only 4 patients, although patients achieving SD were more likely to have a stronger HER2^+^ baseline expression. Pre-clinical TME analysis of CAR-Ms infused into in vivo models showed that CAR-Ms retained their pro-inflammatory phenotype, modulated the TME to a more pro-inflammatory state and induced cross-presentation of antigens from phagocytosed tumor cells to the adaptive immune system.^274^ Correspondingly, translational analyses from the phase I trial showed that CAR-Ms were detected at low frequencies in all but one patient who underwent post-treatment biopsies. Tumor reduction was observed in sites where infiltration with CAR-Ms was predicted from pre-clinical data. Tissue biopsy samples also suggested that the CAR-Ms were able to engage the adaptive immune system through an increase in gene expression associated with T cell activation and exhaustion. This evidence was supported by the discovery of T cells infiltrating tumors from the peripheral circulation, and the expansion of CD8^+^ T cell clones exhibiting an activated cytotoxic phenotype.^278^

Evidence for the suggested pre-clinical mechanism of action of CAR-M monotherapy was thus observed in Phase I despite a lack of durable efficacy. The clinical data suggested three paths forward to boost efficacy. One strategy combines CAR-Ms with checkpoint inhibitors to delay T cell exhaustion and sensitize tumors, examined in a pre-clinical study.^258^ A limited cohort of six patients in the trial received pembrolizumab with CAR-Ms. A second strategy to increase the persistence of CAR-Ms in the TME is to increase cell dose by using monocytes as a starting material for transduction.^330^ A further trial using anti-HER2 CAR monocytes (NCT06254807) has been opened but without published results to date. A third strategy could be to use additional dosing cycles to maintain the immunological pressure on the tumor.

While the requirement in oncology is to engineer cells with a pro-inflammatory phenotype, steering MΦs towards an anti-inflammatory phenotype has also been clinically investigated. MΦs present a therapeutic opportunity in fibrotic diseases such as liver cirrhosis,^331^ where MΦs show phenotypic plasticity during the disease process.^332^

Initial preclinical investigations showed that bone marrow-derived MΦs home to the liver,^333^ promoting regeneration and anti-fibrotic activity in a carbon tetrachloride induced fibrotic mouse model.^334^ Administered MΦs encouraged recruitment of endogenous MΦs and neutrophils, which secreted anti-fibrotic factors MMP-9 and MMP-13 at the site of murine hepatic injury.^334^ The prospect of therapeutic efficacy using un-engineered adoptive transfer of MΦs led to a single-arm Phase 1 academic trial^335^ (ISRCTN10368050) using autologous MΦs manufactured ex-vivo from CD14^+^ monocytes. Nine patients received single escalating doses of up to 1×10^9 cells. The therapy was well tolerated and showed improvements in the MELD score in six out of nine patients. The follow on Phase 2 MATCH study^336^ (ISRCTN10368050) was powered to detect whether MΦs significantly improved the 90-day MELD score between patients randomized to receive MΦs or Standard of Care (SoC).^336^ 27 patients received MΦ therapy, compared to 24 patients receiving SoC; however, the difference between the two groups was not significant. Longer-term follow-up data with patients receiving MΦs showed a statistically significant improvement in overall and transplant free survival.^337^ Difficulty in demonstrating clear efficacy with the primary endpoint could be due to patient selection and behaviour,^336,338^ but also executing a planned strategy of multiple dosing cycles of fresh MΦs, resulting in a single infusion protocol.^336^ There may also be a therapeutic limitation in using non-engineered MΦs to sustain efficacy in more challenging patient segments. Also, without identifiable gene modifications and on-treatment biopsies, it is challenging to track, detect and characterize the phenotype of the autologous cells post-dosing. Addressing these factors, Aleksieva, N. et al. have developed improved cryopreservation and gene modification techniques, and the subsequent product candidate RTX-001 is cryopreserved with multiple doses available from a single patient leukapheresis.^339^ Pre-clinical discovery demonstrated mRNA transfection results in engineered cells retaining better viability and target gene overexpression after cryopreservation compared to pDNA transfection. In vitro testing of gene combinations identified IL-10 and MMP-9 as the optimally identified mRNA payload to boost product phenotype. RTX001 is currently open to enrollment in a commercially sponsored trial from Resolution Therapeutics (NCT06823713).^340^

MΦ therapies have been shown to be safe and well tolerated across different therapeutic areas. Mechanism of action has been shown, but sustained clinical efficacy has not yet been demonstrated. Learnings from nascent clinical and translational data suggest the key to driving therapeutic impact in both the pro- and anti-inflammatory applications of MΦs is threefold. The first factor lies in boosting MΦ cell dose and persistence in the relevant target tissues and second, combining, where appropriate, MΦ therapies with current SoC to provide synergistic benefits.^258^ Third, appropriate clinical trial designs are also an important learning from early studies, in particular the choice of the most appropriate patient inclusion and exclusion criteria and endpoints to best demonstrate clinical utility when faced with heterogenous patient populations.^338^

These challenges are being addressed as clinical development continues in the MΦ field. For example, manufacturing improvements in cell number generation, genetic manipulation and cryopreservation are leading to higher volumes of optimised MΦs that can be administered in multiple dosing cycles.^339^ Other methods of boosting de-facto MΦ doses are also being explored using in vivo CARs^115^ or MΦ-delivered drug payloads.^341,342^ The challenge for the field is to demonstrate meaningful clinical impact, while still ensuring the MΦ modality remains commercially deliverable at scale for future clinical adoption. Readouts from the continued pre-clinical and clinical advancement of MΦ-based therapies, selectively summarised in Table 2, will be vital in elucidating whether MΦ therapies can bridge the gap between proof of mechanism to the demonstration of clinical utility.

**Table 2:** Overview of selected macrophage-based therapeutic candidates and clinical trials

Candidate (or description)	Clinical Phase	Clinical Trial	Sponsor	Strategy	Indication	Status
Allocetra	2	NCT04612413	Enlivex	Allogeneic PBMCs designed to be engulfed by macrophages and reprogram them	Treatment of organ failure in sepsis	Completed
RTX001	1/2	NCT06823713	Resolution Therapeutics	Autologous mRNA engineered macrophages with IL-10 and MMP9	Decompensated liver cirrhosis	Recruiting
(Allogenic macrophages)	1	ISRCTN12637839	University of Edinburgh	Allogeneic alternatively-activated macrophages (AAMs)	Acute liver failure	Recruiting
(CSF2RA gene-corrected macrophages)	1	NCT05761899	Children’s Hospital Medical Center, Cincinnati	CSF2RA gene-corrected macrophages to restore GM-CSF function	hPAP	Recruiting
MT-302	1	NCT05969041	Create Medicines (formerly Myeloid Therapeutics)	Myeloid cell-targeting LNP encoding a TROP2 CAR	Advanced or metastatic epithelial cancers	Recruiting
MT-303	1	NCT06478693	Create Medicines (formerly Myeloid Therapeutics)	Myeloid cell-targeting LNP encoding a GPC3 CAR	HCC and other GPC3 expressing cancers	Recruiting
SIRPant-M	1	NCT05967416	SIRPant Immunotherapeutics	Intratumorally administered autologous SIRPα-low expressing macrophages	Relapsed or refractory NHL	Recruiting
Temferon	1	NCT03866109	Genenta Science	Tumor infiltrating Tie2 expressing monocytes delivering IFN-α to the TME	GBM	Recruiting
PHST001	1	NCT06840886	Pheast Therapeutics	CD24-targetted checkpoint inhibitor to block tumor expressing “don’t eat me” signal	Advanced solid tumors	Recruiting
CUD005	1	NCT06671275	Anhui Provincial Hospital of Chinese Medicine	Autologous macrophages which aim to degrade extracellular matrix and reduce liver scar stasis by secreting a variety of MMPs and anti-inflammatory factors.	Liver cirrhosis	Enrolling by invitation
(anti-HER2 CAR-M)	1	NCT06224738	First People’s Hospital of Hangzhou	Adenoviral vector transduced humanised anti-HER2 CAR-M	HER2^+^ gastric cancer	Not yet recruiting
VTX-0811	Preclinical	-	Verseau Therapeutics	mAb that binds to PSGL-1 and repolarizes macrophages from M2 to M1.	Oncology, indication unknown	IND-enabling
MDC-735	Preclinical	-	Cellis	Macrophages loaded with a ferritin-drug complex to act as a macrophage drug conjugate	GBM, Lung, Uterine and Bladder Cancer	Projected Phase I H2 2025
ReMAST	Preclinical	-	Hermera Pharma	Autologous ex vivo cultured pro-regenerative macrophages to promote nerve injury healing	Spinal Cord regeneration	Projected Clinical Trial by 2027.
AT-06	Preclinical	-	Attralus	Incorporates the PAR (Pan-Amyloid Removal)-peptide as a CAR expressed in macrophages or monocytes		In development
(CAR-M)	Preclinical	-	Inceptor Bio	CAR macrophages with optimised CD47/SIRPα axis to modulate the “don’t eat me” signal	Solid tumors	In development
(iPSC CAR-M)	Preclinical	-	Shoreline Biosciences	iPSC derived gene edited CAR Macrophages	Unknown	In development
MOTO-CAR	Preclinical	-	Thunder Biotech	Nanoparticle in vivo delivery of MSLN, HER2 and HPRT CARs targeting TAMs to induce CAR expression and repolarisation to a pro-inflammatory phenotype	MSLN, HER2 and HPRT expressing tumors	In development
(hALMs)	Preclinical	-	The Hospital for Sick Children	Human stem cells are differentiated into hALMs. ALMs can be delivered directly to the lungs via intratracheal/ intrabronchial administration	Infectious disease e.g. RSV	In development
MT-1000 / MT-2000	Preclinical	-	Macrophage Therapeutics	Conjugated drug payload delivered to modulate macrophages via their CD206 receptor	Unknown	In development

Summary of clinical development stages, trial identifiers, sponsors, therapeutic approaches, target indications, and current development status of selected macrophage (MΦ)-based therapies

## Macrophage signaling cascades: impact on engineering vs. scalable manufacturing

The fundamental challenge of reproducible and cost-effective manufacturing of high-quality cells remains, despite demonstrated pre-clinical and clinical advancements in modulating the signaling of MΦs. With doses in the range of several billion per patient, the necessary numbers are often difficult to achieve with standard 2D culture techniques and require extensive scale-out or scale-up that includes GMP-compatibility and is amenable to automation.

**Fig. 6:**
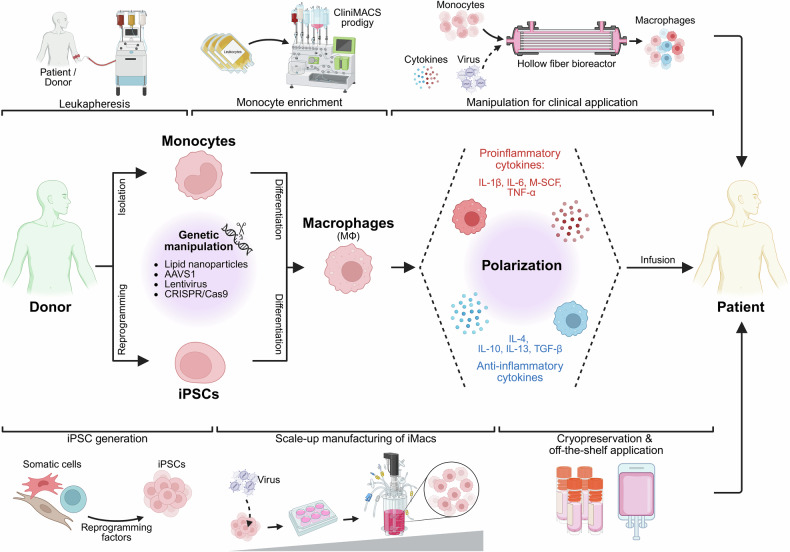
Manufacturing of blood- and iPSC-derived macrophages for cell therapy. Overview of clinical manufacturing workflows for therapeutic MΦs derived from peripheral blood monocytes (top) or iPSCs (bottom). Processes include cell isolation or reprogramming, genetic manipulation, differentiation and polarization, large-scale expansion, and preparation for infusion as autologous or off-the-shelf cell therapy products

The most common source of human MΦs for research and clinical purposes is peripheral blood monocytes and iPSCs. Each has distinct advantages and disadvantages regarding their availability, production and manufacturing characteristics (Fig. 6). Primary monocytes or peripheral blood mononuclear cells (PBMCs), for instance, can be readily extracted from blood products and differentiated towards MΦs with straight forward cytokines. At smaller scales, these usually involve density centrifugation or magnetic selection (either positive or negative), followed by differentiation using major instructive cytokines such as M-CSF or GM-CSF.^343^ GMP-compatible and automated procedures driven by closed system machines, most notably the CliniMACS prodigy from Miltenyi, are available to facilitate clinical scale manufacturing. For example, Fraser et al. used a leukapheresis product containing mononuclear cells and isolated CD14^+^ cells using the CliniMACS prodigy system with microbeads to set up a GMP-compliant process resulting in high sorting efficiency close to 100% and final MΦ yields ranging between 2.9 and 5.7 × 10^8^ cells per patient with further automation steps in the process compared to uses by others.^344^ A similar approach has also been used in other studies for the successful isolation of monocytes, although mostly in the context of dendritic cell generation.^345,346^ Other automated system machines have been used but predominantly for T cell production, like the Cocoon^347^ from Lonza or AMBR from Sartorius.^348^ Donor-to-donor variability can be significant during isolation and differentiation, regarding both quality and quantity of the cells. For example, total nucleated cells isolated from six different donor apheresis bags ranged from 127 × 10^8^ to 234 × 10^8^ cells^349^ with monocyte recovery varying drastically in many cases.^349,350^ The isolated cells can show large differences in their reaction to viruses,^351^ fungal pathogens^352^ and phagocytosis.^353^

The exact isolation method can impact the phenotype of isolated monocytes-derived MΦs^354^ and seemingly minor differences in the production method can influence the transcriptome of the cells, important observations particularly when using synthetic receptor. Regarding autologous use of the cells, it also has to be considered, that patient cells might behave differently to those of healthy controls due to the disease itself, potential comorbidities, genetic predispositions or treatment-related changes. This will be very disease-specific; for example, no difference was found for cells from liver cirrhosis patients,^344,355^ but monocytes from various cancer patients can show phenotypic changes^356^ with cells from breast cancer patients secreting lower levels of IL-1ß, IL-6 and TNF,^357^ but those from renal carcinoma patients show elevated secretion of these cytokines.^358^

Data from iPSC-derived cells remain pre-clinical for now as the field in general is tackling the establishment of large-scale productions and guidelines concerning safety, quality control and formulation of the final end product. iPSC-derived cells offer theoretically an unlimited supply of cells coming from a single clone cell in consistent quality on top of better possibilities of genetic modification of the starting population as well as potential lower cost of goods with a true off-the-shelf solution. Numerous protocols have been developed for generating MΦs from pluripotent stem cells; however, these methods are significantly more complex than the differentiation of primary MΦs. A complete overview detailing the differences of protocols goes beyond the scope of this review and the reader is referred to other summaries.^359^ However, they can be categorized broadly in monolayer cultures, embryoid body (EB)-based protocols and co-cultures that roughly follow the same fundamental phases. First, pluripotent cells undergo mesodermal priming, either through spontaneous differentiation or directed cytokine signaling, mimicking embryonic developmental cues. Cytokines such as bone morphogenetic protein 4 (BMP4) and fibroblast growth factor 2 (FGF2) drive cells toward a mesoendodermal fate, while vascular endothelial growth factor (VEGF) and stem cell factor (SCF) promote hemogenic endothelial formation and support emerging progenitors. For some protocols, the cytokine signaling is replaced to some degree by the co-culture of the cells with for example OP9 cells providing paracrine mediated signaling.^360^ Hematopoietic progenitors are then formed and further supported by, e.g. Interleukin-3^88^ or other hematopoietic cytokines. The last step is myeloid specification of the progenitors and maturation towards MΦs, where the added cytokines mostly align with those used for primary cells. While overall the reported phenotype and functionality of iMacs aligns, direct comparison of protocols is scarce, Klepikova et al. however, found that comparing a EB-dependent spontaneous and EB-independent exogenous factor-dependent protocol the cells showed subtle differences regarding transcriptomic characteristics.^361^ The many protocols published vary regarding the details of the differentiation, for example, the iPSC culture conditions, exact number of cytokines and their concentrations and timings as well as culture conditions. Some of these culture conditions inherently limit how amenable they are for large scale manufacturing, with monolayer protocols restricted to a scale out as well as many of the EB-based protocols relaying on spin EBs.^98,362,363^

Scale-out has been shown to be somewhat feasible with relatively high production of cell numbers. For example, Gutbier et al. managed to scale-out production on to vessels with up to 1000 cm^2^ culture area and combined this with a suspension collection and storage phase generating 70–250 MΦs per iPSC.^362^ These efforts have mostly considered the production of cells in the context of drug screening or similar, as scaling out the production quickly reaches the limitations of how many cells can be produced. Even in the example above it was combined with an easily scalable suspension collection. In this line, it was demonstrated that the process of differentiation of iMacs is also feasible completely in suspension in stirred tank bioreactors (STBR),^83,364^ which are amenable to further scale-up and automation. Here, the cells show a comparable phenotype to those derived from two-dimensional culture as well as/along with functionality in an in vitro phagocytosis assay and in vivo functionality against *P. aerigunosa*.^83^ Even though the differentiation has been established here in suspension, it still contains 2D iPSC culture on feeder cells as well as a small-scale priming step along with not entirely GMP-compatible reagents and manual media changes and harvests. STBR were already adapted to the suspension culture of iPSCs in 3D aggregates^365–367^ and optimized regarding the yield of the cells and a seed train approach which allows continuous culture over multiple passages.^368^ Although some of the published protocols have shown the feasibility of feeder-free and GMP-compatible differentiation,^369^ the main challenge that remains is to bring the individual parts together into one coherent, fully controlled differentiation process. This combination has great potential in fulfilling the criteria needed to bring large-scale production and off-the-shelf availability to patients. An interesting point that remains is the question of ontogeny of the different MΦs used for cell therapy and the potential role it could play in defining their functionality. The overlapping and complex influence of both the ontogeny as well as the tissue environment on MΦs and what role they both play in defining their exact make-up is still a topic for discussion.^370^ Many studies have shown overall broad similarities between PBMC- and iPSC-derived cells regarding phenotype and functionality,^181,371^; however, they have mostly investigated pan-MΦ markers and abilities, with limited in-depth direct comparisons especially in the context of cell therapy. Looking closer, differences become apparent, with most data pointing towards iMacs being derived from primitive hematopoiesis developing in an HSC-independent manner.^83,98,372^ This would make them more akin to tissue-resident MΦ populations, which in many organs develop from early yolk sac or fetal liver progenitors.^4^ Using extensive scRNA seq data for the generation of iMacs in vitro could recapitulate yolk sac hematopoiesis, suggesting iMac development to be independent of a monocyte stage.^372^ Most recently, an angio-hematopoietic progenitor was identified using human iPSCs, highlighting CXCR4 to be relevant in the development of prodefinitive hemato-endothelial cell differentiation and the onset of human iMacs.^373^ To this end, MΦs currently used in first clinical trials are monocyte-derived and thus, come from definitive hematopoiesis, which might call into question whether iPSC-derived MΦs will behave in the exact same way as their primary counterparts or whether the difference in ontogeny will influence their functionality in significant ways. From in vivo data, it remains difficult to separate the spatio-temporal development of the cells from their ontogeny, but it has been shown that cells from various sources can adapt to an empty nicheand fulfill its functional requirements^374^ and are significantly shaped by their microenvironment, including adaptation of subset-specific enhancers,^375,376^ exemplifying the remarkable effect of external force on MΦ identity. However, there are also reports showing that complete convergence might be limited. There are indications of monocyte-derived influx into the liver failing to upregulate TIMD4 as a prominent marker^377^ and hematopoietic progenitor-based repopulation after microglia depletion lacking Sall1,^378^ highlighting a possible lingering influence of the origin of the cells. Other studies have also reported a more direct association between MΦ ontogeny and distinct function as for example given for arterial MΦs under steady state and inflammation^379^ as well as microglia after endotoxin challenge.^380^ A similar observation for the difference in functionality has been observed in the context of trained immunity where recruited monocyte-derived MΦs show distinct differences to (embryonic) tissue-resident cells even after considerable time.^381^ Looking closely, even the heterogeneity in classical monocytes and their origin from either granulocyte-monocyte progenitors or monocyte-DC precursors could contribute to their function and further development into MΦs at least in the murine system.^382^ Our current knowledge of the influence of ontogeny on MΦs is mainly shaped by fate-mapping studies in different mouse models.^370^ There is, however, no dataset directly comparing the persistence and functionality of MΦs from different origins in a therapeutic setting. As such, the influence of the origin of the cells remains an important factor to consider for future cell therapies, but its exact influence remains difficult to assess. Finally, beyond this question, the influence of potential genetic modifications of the final cell product has to be considered in tandem with the ontogeny of the cells. While the origin could affect persistence and functionality, both will likely also be altered by additional modifications of the cells that have the potential to “override” original tendencies.

## Conclusion and lessons learned from early clinical setbacks

In summary, early clinical and preclinical experiences with MΦ and CAR-M therapies have highlighted that sufficient understanding and control of MΦ plasticity is prerequisite for durable (in vivo) efficacy, particularly when considering the highly dynamic and heterogeneous environments in which these cells operate. Ex vivo polarized or naïve MΦs may lose their intended phenotype once exposed to the TME or chronically inflamed tissues, where competing cytokine gradients, stromal interactions, and metabolic constraints actively reshape cellular behavior. For instance, IFN-γ/LPS-activated pro-inflammatory MΦs and early CAR-M products showed only transient anti-tumor activity, but were progressively coerced into pro-tumor-like, immunosuppressive states, limiting clinical benefit despite tolerable safety. This phenotypic drift highlights the dominant influence of the local microenvironment over engineered programming. Similarly, studies in fibrotic disease using unmodified autologous MΦs demonstrated biological activity and favorable tolerability, yet only modest and heterogeneous clinical responses, consistent with incomplete or unstable in vivo reprogramming. Together, these findings collectively highlight that MΦ plasticity, while therapeutically-attractive, should not only be viewed as an opportunity, but also remains a major hurdle to sustained efficacy if not actively engineered and controlled. These outcomes further suggest that directly transferring “T cell logic” to myeloid cells, such as simple CD3ζ-based CAR designs with minimal consideration of myeloid signaling, does not sufficiently account for the complexity of MΦ biology. Unlike T cells, MΦs integrate signals through a broad spectrum of innate immune receptors and transcriptional networks, leading to fundamentally different activation dynamics. Key variables, including MΦ ontogeny (monocyte-derived versus iPSC-derived, TRM-like), tissue imprinting, metabolic status, and prior “training” of MΦs into highly dynamic activation states, remain insufficiently explored, despite evidence that they critically influence persistence, functionality, and therapeutic durability. Thus, future avenues should prioritize systematic investigation of these factors, as current approaches largely overlook this layered regulation, treating MΦ sources as interchangeable and assuming that a single CAR construct can override local cues. In reality, ontogeny and microenvironment jointly shape persistence, trafficking, threshold for re-polarization, and crosstalk with T- and NK cells. In addition, disease-specific factors such as hypoxia, nutrient deprivation, and chronic inflammation as well as ECM stiffness and stromal interactions, further modulate MΦ behavior in vivo.

Going forward, rational design of CAR-Ms and other genetically engineered MΦs must be grounded in a deeper, indication-specific understanding of MΦ heterogeneity. This includes systematic mapping of disease-associated MΦ states, high-throughput screening of MΦ-centric signaling domains and payloads (e.g. MyD88/SYK modules, cytokine circuits, dominant-negative receptors), and deliberate choice of cell sources based on desired function and longevity. Integration of single-cell and spatial omics technologies will be key to identifying targetable MΦ states and niche-specific vulnerabilities. Coupling these engineering strategies with biomarker-driven patient selection and rational combinations (e.g. checkpoint blockade, TGF-β/CXCR4 inhibition, metabolic modulation) will be essential to stabilize pro-therapeutic MΦ states in vivo. Such combinatorial approaches may also help overcome resistance mechanisms that limit monotherapy efficacy. Only by aligning CAR and synthetic biology approaches with the intrinsic plasticity and context dependence of MΦs can the field move from proof-of-mechanism to reproducible clinical efficacy. Importantly, integration of systems immunology, single-cell data, and computational modeling will further improve prediction and control of MΦ behavior in different disease settings.

In addition, long-term challenges including off-the-shelf availability, cost efficiency, and donor-to-donor variability may further restrict widespread adoption. iPSC-derived therapies offer a promising alternative by providing a true off-the-shelf solution. Yet, significant hurdles remain, including scalability, GMP-compliant manufacturing, cryopreservation, and immune cloaking. While substantial progress has been made, further advancements are essential to ensure that iPSC-derived cells can be produced in sufficient quantity and quality to meet clinical demands. Standardized differentiation protocols, robust quality control, and careful regulatory oversight, including monitoring long-term persistence, potential off-target effects, and unintended immune activation, will be essential for clinical translation. Finally, important lessons can be drawn from early clinical setbacks. These include the need to better anticipate MΦ reprogramming in vivo, to design constructs that are resilient to suppressive microenvironments, and to move beyond simplified polarization paradigms toward a deeper understanding of MΦ activation states.

Beyond the optimization of MΦs as stand-alone therapeutic products, future strategies may increasingly exploit their function as a cellular complement within tandem cell therapy concepts. MΦs are ideally positioned to cooperate with other immune effector cells, as they can remodel tissue environments, promote antigen presentation, secrete instructive cytokines, clear debris or tumor cells by phagocytosis, and shape subsequent adaptive and innate immune responses. Combining engineered MΦs with CAR-T cells, T cells, NK cells, or CAR-NK cells could therefore provide synergistic activity, particularly in complex tissues such as solid tumors, fibrotic organs, or chronically-inflamed sites, where single-cell modalities often fail to overcome suppressive microenvironments. In such settings, MΦs may act as tissue-conditioning and immune-orchestrating cells that enhance lymphocyte recruitment, persistence, and effector function, while T- or NK cells provide potent cytotoxic activity. These tandem-cell approaches could also be designed bidirectionally, with lymphocytes supporting MΦ activation and MΦs stabilizing local immune remodeling, thereby expanding the therapeutic potential of combinatorial cell therapy platforms.

Taken together, while MΦ-based therapies hold considerable promise, their successful clinical implementation will depend on embracing the inherent complexity of MΦ biology and translating this knowledge into more sophisticated and context-aware therapeutic designs. Looking ahead, continued convergence of immunology, synthetic biology, and bioengineering is likely to enable next-generation MΦ therapies with improved precision, durability and clinical impact.
